# *Mmp12* Is Upregulated by *in utero* Second-Hand Smoke Exposures and Is a Key Factor Contributing to Aggravated Lung Responses in Adult Emphysema, Asthma, and Lung Cancer Mouse Models

**DOI:** 10.3389/fphys.2021.704401

**Published:** 2021-11-29

**Authors:** Alexandra Noël, Zakia Perveen, Rui Xiao, Harriet Hammond, Viviana Le Donne, Kelsey Legendre, Manas Ranjan Gartia, Sushant Sahu, Daniel B. Paulsen, Arthur L. Penn

**Affiliations:** ^1^Department of Comparative Biomedical Sciences, School of Veterinary Medicine, Louisiana State University, Baton Rouge, LA, United States; ^2^Department of Anesthesiology, Columbia University Medical Center, New York, NY, United States; ^3^Finn Pathologists – CVS, Harleston, United Kingdom; ^4^Department of Pathobiological Sciences, School of Veterinary Medicine, Louisiana State University, Baton Rouge, LA, United States; ^5^Department of Mechanical and Industrial Engineering, Louisiana State University, Baton Rouge, LA, United States; ^6^Department of Chemistry, University of Louisiana at Lafayette, Lafayette, LA, United States

**Keywords:** second-hand smoke, *in utero* exposures/prenatal exposures, *Mmp12*, emphysema, asthma, lung cancer

## Abstract

Matrix metalloproteinase-12 (*Mmp12*) is upregulated by cigarette smoke (CS) and plays a critical role in extracellular matrix remodeling, a key mechanism involved in physiological repair processes, and in the pathogenesis of emphysema, asthma, and lung cancer. While cigarette smoking is associated with the development of chronic obstructive pulmonary diseases (COPD) and lung cancer, *in utero* exposures to CS and second-hand smoke (SHS) are associated with asthma development in the offspring. SHS is an indoor air pollutant that causes known adverse health effects; however, the mechanisms by which *in utero* SHS exposures predispose to adult lung diseases, including COPD, asthma, and lung cancer, are poorly understood. In this study, we tested the hypothesis that *in utero* SHS exposure aggravates adult-induced emphysema, asthma, and lung cancer.

**Methods:** Pregnant BALB/c mice were exposed from gestational days 6–19 to either 3 or 10mg/m^3^ of SHS or filtered air. At 10, 11, 16, or 17weeks of age, female offspring were treated with either saline for controls, elastase to induce emphysema, house-dust mite (HDM) to initiate asthma, or urethane to promote lung cancer. At sacrifice, specific disease-related lung responses including lung function, inflammation, gene, and protein expression were assessed.

**Results:** In the elastase-induced emphysema model, *in utero* SHS-exposed mice had significantly enlarged airspaces and up-regulated expression of *Mmp12* (10.3-fold compared to air-elastase controls). In the HDM-induced asthma model, *in utero* exposures to SHS produced eosinophilic lung inflammation and potentiated *Mmp12* gene expression (5.7-fold compared to air-HDM controls). In the lung cancer model, *in utero* exposures to SHS significantly increased the number of intrapulmonary metastases at 58weeks of age and up-regulated *Mmp12* (9.3-fold compared to air-urethane controls). In all lung disease models, *Mmp12* upregulation was supported at the protein level.

**Conclusion:** Our findings revealed that *in utero* SHS exposures exacerbate lung responses to adult-induced emphysema, asthma, and lung cancer. Our data show that MMP12 is up-regulated at the gene and protein levels in three distinct adult lung disease models following *in utero* SHS exposures, suggesting that MMP12 is central to *in utero* SHS-aggravated lung responses.

## Introduction

Chronic obstructive pulmonary diseases (COPD), asthma, and lung cancer are leading public health care burdens for which therapeutic options are currently limited. In the United States (U.S.), COPD prevalence – 15 million people – results in an annual estimated healthcare cost of $50 billion. Asthma prevalence – 25.7 million people, including 7 million children – carries a yearly healthcare cost of $56 billion (Guarascio et al., 2013; Wheaton et al., 2015; Nunes et al., 2017). Lung cancer, the deadliest type of cancer in the U.S., has an estimated annual health care cost of $13.4 billion (Park and Look, 2019). Emphysema, a significant COPD, and asthma are both complex lung diseases arising from gene-environment interactions, characterized by chronic lung inflammation, mucus hypersecretion, airflow obstruction, and airway remodeling. While these two lung structural conditions show convergence of symptoms and clinical features, asthma is partly reversible, while emphysema is not (Abramson et al., 2014). Further, although cigarette smoking is the leading cause of COPD and lung cancer, only a minority of smokers develop these diseases, whereas *in utero* exposures to cigarette smoke (CS) and second-hand smoke (SHS) are associated with asthma development in those offspring. Established environmental risk factors for both COPD and asthma include early life exposures to CS and SHS (Abramson et al., 2014). Although more than 7,000 lung cancer deaths among American nonsmokers are attributed to SHS exposure (Max et al., 2012), the link between *in utero* SHS exposures and adult lung cancer remains unclear. Currently, at least 12% of women smoke during their pregnancies (Tong et al., 2009; Scherman et al., 2018), resulting in the birth of approximately 400,000 infants involuntarily exposed to CS *in utero* each year in the US (Rehan et al., 2009). That is in addition to the 126 million Americans exposed annually to SHS, including non-smoking pregnant women (Menzies, 2011) and the 15 to 18% of children exposed annually in American households. Most importantly, the number of deaths related to active smoking and SHS exceeds 480,000 annually, and estimates of the societal direct medical cost incurred from SHS exposures range from $50 to 181 million annually (Max et al., 2012; Mason et al., 2015). Thus, these data emphasize the critical need to investigate *in utero* SHS exposures in adult lung diseases.

The causal association between maternal smoking and asthma in children was first published in 1975 (de Haas, 1975). Subsequently, associations between *in utero* SHS and asthma development in the offspring also were established (Zacharasiewicz, 2016). Work from our laboratory showed that *in utero* SHS exposure aggravated ovalbumin-induced asthma in adult mice, in terms of declines in lung function and increased eosinophilic lung inflammation (Penn et al., 2007; Rouse et al., 2007; Xiao et al., 2013). While the significant contribution of maternal smoking to the rise of asthma worldwide is well documented, there is increasing evidence that *in utero* SHS exposures also may be associated with COPD (Eisner et al., 2010; Zacharasiewicz, 2016). Early-life exposure to SHS has been related to early adulthood development of emphysema (Lovasi et al., 2010). Earlier age onset of emphysema is supported by our data from mouse studies where *in utero* SHS significantly down-regulated the expression of Serpina1a, the mouse orthologue of α1-antitrypsin and a known genetic risk factor for emphysema development, at both the gene and protein levels (Noël et al., 2017). Epidemiological and experimental data from decades ago show that *in utero* exposures to CS result in long-term adverse effects on lung maturation and function. These effects include a significant decrease in alveolarization and reduced air saccules (Bassi et al., 1984; Collins et al., 1985), and decreased lung surface area, lung capacities, and volumes (Hanrahan et al., 1992; Cunningham et al., 1994; Gilliland et al., 2000). All that is in addition to increased risk of pulmonary infections (Strachan and Cook, 1997), asthma (Strachan and Cook, 1998; von Mutius, 2002), and COPD in the offspring (Coultas, 1998; Le Souëf, 2000; Rehan et al., 2009; Rennard and Drummond, 2015). Since it crosses the human placenta and accumulates in the fetal respiratory tract, nicotine is thought to play a crucial role in these altered lung development processes (Hylkema and Blacquiere, 2009; Rehan et al., 2009). While associations between *in utero* SHS exposures, asthma, and emphysema have been demonstrated both epidemiologically and experimentally, little is known about possible shared mechanisms by which *in utero* SHS aggravates adult lung diseases including emphysema, asthma, and lung cancer.

CS induces a persistent activation of lung macrophages, which produce matrix metalloproteinase 12 (*Mmp12*; Xu et al., 2017). *Mmp12* overexpression dysregulates homeostasis of the extracellular matrix by degrading alveolar elastin and contributes to the destruction of alveolar walls in human lungs, as well as in *in vivo* models (Mukhopadhyay et al., 2010; Xu et al., 2017). *Mmp12* is part of a family of 24 structurally and functionally-related zinc-dependent extracellular matrix proteolytic enzymes that have multiple well-documented physiological functions, including tissue remodeling, regeneration, growth, and repair; as well as pathological roles, involving degradation of the extracellular matrix (ECM) and basement membrane (Elkington and Friedland, 2006). Several studies have shown that *Mmp12* plays a role in emphysema development in humans. *Mmp12* overexpression in the lungs is associated with reduced lung diffusing capacity (Molet et al., 2005; Chaudhuri et al., 2012; Ishii et al., 2014), as well as increased levels of *Mmp12* in the sputum of COPD patients (Demedts et al., 2006; Elkington and Friedland, 2006). These clinical results are supported by experimental data demonstrating (A) that elastase causes lung tissue destruction and (B) that mice deficient in *Mmp12* are resistant to CS-induced emphysema (Hautamaki et al., 1997; Selman et al., 2003; Morse and Rosas, 2014; Shibata et al., 2018). In addition, a genetic polymorphism in *Mmp12* in humans is associated with COPD and asthma (Di Valentin et al., 2009; Mukhopadhyay et al., 2010; Bagdonas et al., 2015). Regarding asthma, *Mmp12* gene knockout mice also exhibit reduced lung inflammation following cockroach antigen-induced asthmatic responses (Mukhopadhyay et al., 2010; Morse and Rosas, 2014). In mice, *Mmp12* is modulated throughout the stages of asthma pathology, and its overexpression persists over time (Di Valentin et al., 2009). Moreover, since MMPs are involved in ECM degradation, they may play crucial roles in the tumor microenvironment by promoting tumor invasion and metastasis (Qu et al., 2009). *MMP12* protein is overexpressed in lung cancer and correlates with local recurrence and metastasis, as well as with early cancer-related deaths in non-small cell lung cancer patients (Kettunen et al., 2004; Hofmann et al., 2005; Sun et al., 2006; Tian et al., 2015). Those findings are supported by experimental data where knockdown of *MMP12* in a lung adenocarcinoma cell line inhibited the growth and invasiveness of these cells (Lv et al., 2015). Overall, *Mmp12* in the lungs plays critical roles in acute pro-inflammatory allergen responses, in chronic airway remodeling, and in ECM degradation, highlighting its involvements in emphysema, asthma, and lung cancer pathogenesis (Mukhopadhyay et al., 2010; Xu et al., 2017; Shibata et al., 2018).

Long-term compromised lung function will likely have an adverse effect on the respiratory system during childhood, adolescence, and adulthood. Previously, we reported that *in utero* exposures to SHS alone significantly altered lung structure and produced lung function decline in mice (Noël et al., 2017). These effects, with minor–moderate immediate impact, might not translate directly into a disease; rather, they suggest a pre-disease state, where already compromised lungs may be more vulnerable and thus less resistant, to environmental stressor exposures later in life. The long-term effects of living with physiologically compromised lungs on the development of adult chronic lung diseases are largely unknown. A common mechanism by which *in utero* SHS affects emphysema, asthma, and lung cancer, three distinct lung diseases, is as yet to be determined. Since *Mmp12* production is induced by CS (Churg et al., 2003) and is involved in atypical ECM proteolysis in several lung diseases (Lagente et al., 2005, 2009; Greenlee et al., 2007), it is plausible that *in utero* SHS exposures, similarly to what is observed in adult CS exposures (Hautamaki et al., 1997; Churg et al., 2003), up-regulate *Mmp12* expression, leading to exacerbated dysregulation of ECM remodeling after exposure to a second environmental risk factor. To our knowledge, there are no studies to date investigating the role(s) *Mmp12* play(s) in *in utero* SHS exposure-promoted adult lung diseases. To address that here, we used a highly controlled and reproducible *in utero* SHS mouse exposure model coupled with well-characterized mouse models of either elastase-induced emphysema, house-dust mite (HDM)-induced asthma, or urethane-induced lung cancer. In addition, current epidemiological evidence, in contrast to earlier reports, shows that COPD mortality in women is higher than in men (Dransfield et al., 2007; Camp et al., 2009). It is also well-established that adult asthma is more prevalent in females than in males, whereas the opposite is true for childhood asthma (de Marco et al., 2000). Moreover, lung cancer incidence was shown recently to be higher among young women than young men, a phenomenon that was not entirely explained by differences in sex-specific smoking behaviors (Jemal et al., 2018; Fidler-Benaoudia et al., 2020). Thus, based on the current literature (de Marco et al., 2000; Carey et al., 2007; Dransfield et al., 2007; Camp et al., 2009; Jemal et al., 2018; Fidler-Benaoudia et al., 2020) including our previously published results, we elected to conduct the lung disease outcome experiments described here in female mice.

## Materials and Methods

### Overall Experimental Design

**Figure 1:**
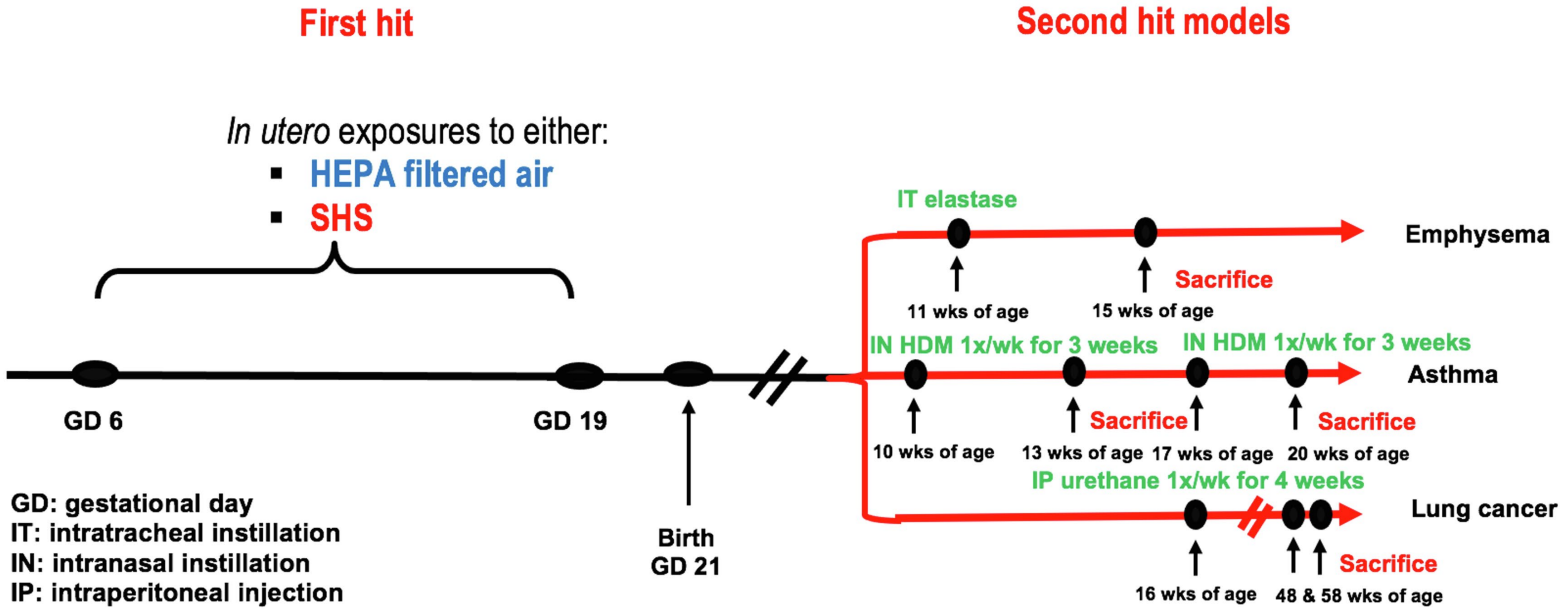
Experimental study design. First hit model: *in utero* air or SHS exposures from gestational days 6 to 19. Second hit models: elastase-induced emphysema, house-dust mite-induced asthma, and urethane-induced lung cancer in adult female offspring mice.

Pregnant BALB/c mice were exposed from gestational days 6–19 to either 3 or 10mg/m^3^ of SHS or to filtered air. At 10, 11, 16, or 17weeks of age, female offspring were treated with either elastase to induce emphysema, HDM to initiate asthma, or urethane to induce lung cancer. At sacrifice, specific disease-related lung responses were assessed, including lung function, inflammation, as well as gene and protein expression. The experimental study design is illustrated in Figure 1.

### *In utero* SHS Exposures – First Hit

In this study, 10–12-week-old pregnant BALB/c mice (Harlan/Envigo, Indianapolis, IN) were exposed to either HEPA filtered air or to a surrogate for SHS, composed of 100% sidestream smoke diluted with HEPA-filtered air, as described (Xiao et al., 2012, 2013; Noël et al., 2017). Mice were exposed in 1.3m^3^ dynamic inhalation chambers for 4h per day from gestational days 6 to 19. SHS was generated by a 10-port cigarette smoke generator (TSE Systems, Bad Homburg, Germany) using standardized 3R4F research cigarettes (University of Kentucky, Lexington, KY). The targeted total particulate matter (TPM) concentrations for the SHS exposures were 3 or 10mg/m^3^. The TPM mass concentration was determined gravimetrically on filters (25mm hydrophilic glass fiber filter with a 0.7μm pore size; Millipore Sigma, Cat. #AP4002500, Burlington, MA) placed in cassettes. TPM samples were collected at a flow rate of 3L/min for subsequent gravimetric analysis. The SHS exposures also were monitored in real-time by a DustTrak Aerosol Monitor 8520 (TSI Inc., Shoreview, MN). Carbon monoxide (CO) concentrations were measured by a *MIRAN SapphIRe* portable infrared ambient spectrometer (ThermoFisher, Franklin, MA), as described previously (Penn et al., 2007; Noël et al., 2017). Tables 1–3 show the characterization of the SHS and control HEPA-filtered air exposures for each of the three studies. From birth to humane euthanasia, mice were maintained on HEPA-filtered air-ventilated racks. Mice were housed and handled in accord with the NIH Guide for the Care and Use of Laboratory Animals. All procedures and protocols were approved by the Louisiana State University Institutional Animal Care and Use Committee.

**Table 1:** *In utero* SHS+elastase – Emphysema study.

Parameters	*In utero* AIR	*In utero* 3R4F 10mg/m^3^ SHS
Mass concentration (mg/m^3^)^a^ ±SD	–	10.61±1.8
CO concentration range (PPM)	–	22.9–30.4
Temperature (°C)±SD	21.63±1.3	22.6±1.3
Relative humidity (%)±SD	43.56±8.2	43.5±8.7

*SD, standard deviation*.

a *Average mass concentration determined gravimetrically*.

**Table 2:** *In utero* SHS+HDM – Asthma study.

Parameters	*In utero* AIR	*In utero* 3R4F 3mg/m^3^ SHS
Mass concentration (mg/m^3^)^a^ (± SD)	–	3.14±1.0
CO concentration range Min–Max (PPM)	–	1.39–13.05
Temperature (°C)±SD	23.97±0.2	23.9±0.3
Relative humidity (%)±SD	39.81±7.0	49.8±14.8

*SD, standard deviation*.

a *Average mass concentration determined gravimetrically*.

**Table 3:** *In utero* SHS+urethane – Lung cancer study.

Parameters	*In utero* AIR	*In utero* 3R4F 10mg/m^3^ SHS
Mass concentration (mg/m^3^)^a^ (± SD)	–	10.02±2.4
CO concentration range Min–Max (PPM)	–	6.32–33.6
Temperature (°C)±SD	23.97±0.2	23.77±0.2
Relative humidity (%)±SD	39.81±7.0	42.04±6.2

*SD, standard deviation*.

a *Average mass concentration determined gravimetrically*.

### Second-Hit Models

#### Elastase-Induced Emphysema

At 11weeks of age, female offspring were instilled intratracheally with either 1× PBS (controls; 50μl) or 0.1U/g of porcine pancreatic elastase (Millipore Sigma, Cat. #324682, Burlington, MA) suspended in 1× PBS (injection volume: 40–50μl based on mouse body weight). At 15weeks of age lung function was evaluated. Immediately after sacrifice (15weeks of age), inflammatory responses, airspace enlargement, as well as gene and protein expression were assessed.

#### HDM-Induced Asthma

At 10 or 17weeks of age, female offspring were instilled intranasally, 1×/week for 3 consecutive weeks, with either 1× PBS (controls; instillation volume: 30μl: 15μl per nostril) or with 25μg of HDM (D. pteronyssinus, Stallergenes Greer, cat. # XPB7OD3A2.5, Lenoir, NC) suspended in 1× PBS (instillation volume: 30μl: 15μl per nostril). At 13 and 20weeks of age, we evaluated lung function, broncho-alveolar lavage fluids (BALF), lung tissue histopathology, as well as gene and protein expression.

#### Urethane-Induced Lung Cancer

From 16 to 19weeks of age, female offspring were treated weekly with a intraperitoneal injection of either 1× PBS (controls; 0.3ml) or urethane (ethyl carbamate: 1g/kg; Millipore Sigma, Cat. # U2500, St-Louis, MO) suspended in 1× PBS (injection volume: 0.23–0.3ml based on mouse body weight). Urethane is a Group 2A carcinogen (Kwon and Berns, 2013). Groups of mice were sacrificed at 48 and 58weeks of age. BALF was collected for assessment of inflammatory responses and lung tissue was used for gene and protein expression. Tumors were analyzed for number, size, volume, and histopathological classification.

In addition, for the lung cancer study, we recorded the mouse body weights and lung weights and calculated the lung organ index as: lung organ index=weight of lung (g)/body weight (g)×100. We also sized the visible lung surface tumors with an electronic digital caliper (Fisher Scientific) that has a measuring range from 0 to 200mm and an indication of an error of 0.03mm. Tumors were estimated to be either spherical or non-spherical. For spherical tumors, the volume was calculated as: volume=4/3 π r^3^; and for non-spherical tumors, the volume was calculated as: volume=0.52 ab^2^ (a=larger and b=smaller diameter).

### Pulmonary Function Testing – *FlexiVent* – For Emphysema and Asthma Studies

Mouse pulmonary function testing was performed as described (Noël et al., 2017, 2020) on the day of sacrifice by forced oscillation techniques (FOT) with the *flexiVent* system (SCIREQ, Montreal, Canada). Briefly, mice were anesthetized by subcutaneous injection of a ketamine/xylazine cocktail, tracheostomized, and then connected to the *flexiVent* system. Respiratory system resistance (Rrs) and compliance (Crs) were measured using pre-defined scripts from the *flexiVent* system. At least five measurements for each parameter were recorded and averaged per mouse. Mice also were exposed to incremental doses of 0, 12.5, 25, and 50mg/ml of methacholine before sacrifice. Measurements were accepted only if the coefficient of determination was >0.95, assuring the fit of the single-compartment model. Following the procedure, mice were euthanized by an intraperitoneal injection of Beuthanasia-D (Merck Animal Health; Kenilworth, NJ).

### Broncho-Alveolar Lavage Fluid Collection – For Emphysema, Asthma, and Lung Cancer Studies

Following euthanasia, the lungs of each non-flexiVented mouse were lavaged twice with 0.5ml of phosphate-buffered saline (PBS). The BALF was kept on ice and was later centrifuged to separate the BALF cell pellet from the BALF supernatant. The BALF supernatant was stored at −80°C for subsequent analyses, while the cell pellet was resuspended in 150 μl of BALF and used to prepare slides for differential cell counts (300 cells). Smears were stained with a modified Wright’s stain.

### Histopathology Analysis of Lungs – For Emphysema, Asthma, and Lung Cancer Studies

For each non-flexiVented mouse, as previously described (Noël et al., 2017, 2020), we inflated and pressure-fixed (25cm) the lungs with buffered formalin (10%) *via* intratracheal instillation. We sectioned and stained 5-mm thick lung sections, as described previously (Xiao et al., 2013; Noël et al., 2016, 2017). This was followed by H&E staining on 5-mm thick paraffin-embedded tissue sections.

For the asthma study, slides containing sections of at least 3 lobes of the right lungs from at least 9 mice per group were evaluated by a board-certified veterinary pathologist. All slides were evaluated in detail to determine the gradient of changes relative to the following lung parameters: peribronchial eosinophils, peribronchial neutrophils, peribronchial lymphocytes, alveolar macrophages, alveolar polymorphonuclear cells, Mott cells, tissue damage and mucous metaplasia. The values for these scores are as follows:

Overall inflammation score was 0=normal, 1=detectable at 100×, 2=obvious at 20× localized, 3=obvious at 20× widespread, 4=complete bronchial cuffs localized, 5=complete bronchial cuffs widespread.

All other parameters were scored: 0=normal. 1=minimal, 2=mild, 3=moderate, 4=severe.

For the lung cancer study, all slides were evaluated in detail to determine the gradient of changes relative to the following parameters in the lungs, as per Ron A. Herbert (personal communication; Nikitin et al., 2004; Pandiri, 2015).

#### Atypical Hyperplasia

Focal, nodular, proliferative lesions, 2mm–1cm in diameter; alveolar architecture is maintained; no cellular atypia.

#### Alveolar/Bronchiolar Adenoma

Solid, papillary, or pseudoalveolar growth patterns; alveolar architecture is distorted or effaced; no to minimal cellular pleomorphism. Mitotic rate is low. Compression without invasion of surrounding tissue.

#### Alveolar/Bronchiolar Carcinoma

Irregular variable patterns of growth with obliteration of normal architecture; cuboidal to columnar, pleomorphic cells with atypia. Multilayered epithelia. Parenchymal invasion, and potential metastases. Increased nuclear to cytoplasmic ratio. May be multiple nuclei and nucleoli. Increased mitotic rate.

#### Intrapulmonary Metastasis

Much smaller than the other tumors in the lung, separated from other tumors in the lung, clearly malignant; vascular association.

### Lung Morphometric Analysis – For Emphysema Study

Morphometric analysis was performed on the lung slides prepared for histopathological evaluation, as detailed above. Briefly, as described previously (Noël et al., 2017, 2020), a grid line system with 16 (4×4), horizontal lines was overlaid on each 10× lung micrograph. The mean linear intercept (MLI, or Lm) and surface are per unit volume (SApUV) were calculated based on the number of times a line intercepts and alveolar wall (Xiao et al., 2016). At least five images of different lung sections per mouse were analyzed. Images were obtained with a Nikon Eclipse E400 microscope (Nikon, Tokyo, Japan).

### Lung Harvest and mRNA Extraction – For Emphysema, Asthma, and Lung Cancer Studies

For each non-flexiVented mouse, as previously described (Xiao et al., 2012, 2013; Noël et al., 2017, 2020), a small piece from the right superior lung lobe was collected, stored in RNA later, and subsequently used for total mRNA extraction, *via* the RNeasy Plus Mini Kit (Qiagen; Germantown, MD), per manufacturer instructions. RNA sample quantity and purity assessment were evaluated by a NanoDrop ND-1000 Spectrophotometer (NanoDrop, Wilmington, DE). RNA samples were assayed in 1:5 dilutions with an Agilent 2100 BioAnalyzer and Agilent RNA 6000 Nano Series II Kits (Agilent Technologies, Palo Alto, CA).

### Gene Expression Analysis by Quantitative RT-PCR – For Emphysema and Asthma Studies

Quantitative real-time PCR (qRT-PCR) was performed on cDNA samples of lung homogenates, as described (Noël et al., 2020). We used inventoried TaqMan Gene Expression Assays primer-probe sets (Applied Biosystems) and an Applied Biosystems 7300 Real-Time PCR System to evaluate the expression of selected genes. For the asthma study, a total of 24 genes were analyzed by RT-PCR, while for the emphysema study a total of 8 genes were analyzed by RT-PCR. For each gene, we used reaction volumes of 25 μl and 40 reaction cycles. We used the comparative cycle threshold (ΔΔC_T_) method to determine relative gene expression, with each gene normalized to hypoxanthine-guanine phosphoribosyltransferase (*Hprt1*) expression. Results are reported as fold-change in treated samples compared to controls (2^−ΔΔCT^).

### Gene Expression Analysis by RT^2^ Profiler PCR Array – For Lung Cancer Study

The lungs of the mice from the lung cancer study were analyzed for the expression of 84 genes related to lung cancer (PAMM-134Z) on a mouse RT^2^ PCR array (Qiagen, cat. #330231), per manufacturer’s instructions. We used the RT^2^ First Strand Kit (Qiagen cat. #330401) to reverse-transcribe 0.5 μg of total RNA. We subsequently diluted the cDNA with RNase-free water, as previously described (Noël et al., 2020). Then, the RT^2^ SYBR Green qPCR Master Mix (Qiagen Cat. #330503) was mixed with the cDNA samples. The PCR Array plate contains the pre-dispensed gene-specific primer sets. We added 25 μl aliquots to each well. We used an Applied Biosystems 7300 Real-Time PCR System to evaluate the expression of those genes. The Qiagen PCR Array data analysis software was used to calculate the gene expression and fold-change by the ΔΔCt method.

### Gene Expression Analysis by RNA-Sequencing – For Emphysema Study

We evaluated global gene expression in lung homogenates of individual mice (*n* =4 per group) from the emphysema study. Samples for RNA sequencing were processed and sequenced by the company formally called Expression Analysis (Morrisville, NC), as described (Noël et al., 2017). RNA sequencing was performed on an Illumina sequencing platform by HiSeq – 2x50bp-PE sequencing. A median of 16,466 genes for our mouse lung transcriptome was detected. Subsequently, bioinformatics analyses were conducted. We considered gene probes with at least 2-fold up-/down-regulation (*p* <0.05) and false discovery rate (FDR)<0.05 to be differentially expressed.

### Quantification of Lung Collagen by Multiphoton Imaging – For Lung Cancer Study

Using unstained lung slides, prepared as described above, lung fibrillar collagen content was measured and imaged using second harmonic generation (SHG) microscopy. SHG and two-photon excited fluorescence imaging were carried out using a Leica SP5 resonant scanning multiphoton confocal inverted microscope (Leica Microsystems), coupled to a Spectra Physics Mai-Tai tunable pulsed near-IR laser (690nm–1,040nm). All SHG imaging experiments were performed with an excitation wavelength of 860nm with a laser pulse width of 70fs produced at an 84MHz repetition rate. The laser pulses were focused onto lung tissue specimens through a 20×, 0.70 NA air objective (Obj. HC PL APO 20×/0.70 CS, Leica) for noncontact imaging mode. The propagating SHG emission was collected in the backward direction using the same objective by selecting a filter cube (680nm Short Pass filter) equipped with 320–430nm range band pass filter (>90% transmission). The two-photon fluorescence signals from tissue were collected through a 486–506nm narrow range band-pass filter and detected by non-descanned modular type photomultiplier tube (PMT) detectors. In SHG imaging, the typical laser power measured at the objective focus is about 50mW. LAS X software (Leica Microsystems) was employed for laser scanning control and image acquisition. Image sizes were 1,024×1,024 pixels captured at a 400Hz scan speed per line resulting in pixel dwell time of 2.44μs per pixel. SHG and two-photon excited fluorescence are represented in pseudo color, green and red, respectively. SHG image analyses were performed using ImageJ software (NIH, Bethesda, MD) employing the image segmentation and simple threshold pixel counting methods. For each mouse, three different lung sections were imaged by SHG microscopy.

### Extraction of Proteins – For Emphysema, Asthma, and Lung Cancer Studies

For the non-flexiVented mice, a small piece from the right superior lung lobe was collected, directly frozen on dry ice, stored at −80°C, and subsequently used for protein extraction, as previously described (Noël et al., 2017). Briefly, we mechanically broke lung tissues snap-frozen in liquid nitrogen, and then quickly transferred the samples to micro-centrifuge tubes containing 300μl of RIPA lysis buffer (Santa Cruz Biotechnology, Dallas, TX, United States) and three 2.5mm zirconia/silica beads (Biospec Products Inc.). We used a Tissue Lyser II (Qiagen, Germantown, MD, United States) set at 25MHz for 2min to completely lyse the tissue. Afterward, the samples were centrifuged at 13,000g for 10min at 4°C. We used the BCA protein assay kits (Thermo Scientific, Waltham, MA, United States) to determine the protein concentrations in the supernatants.

### MMP12 Proteins Analysis – For Emphysema, Asthma, and Lung Cancer Studies

We used a MILLIPLEX Mouse MMP Magnetic Bead Panel 2 - Immunology Multiplex Assay (cat# MMMP2MAG-79K, Millipore Sigma, St-Louis, MO) to determine the lung tissue protein concentrations of MMP12. The assay was run according to the manufacturer’s protocol. Twenty-five μg of protein from each supernatant were added to each well of the assay plate. The assays use antibodies linked to magnetic beads, and the relative concentration of each sample analyzed is compared with the concentrations of the standard controls provided by the manufacturer. Each sample was assessed in duplicate. The multiplex assay was run on the Bio-Plex 200 system (Bio-Rad).

### Statistical Procedures – For Emphysema, Asthma, and Lung Cancer Studies

All biological endpoints, including BALF cytology, lung function, and lung morphometry, were analyzed by the Students’ *t*-test for pairwise comparisons, or by ANOVA followed by the Tukey’s test for multiple comparisons. Data are expressed as mean±standard error of the mean (SEM). Results were considered statistically significant at *p* <0.05. We carried out statistical analyses with the Statistical Package for the Social Sciences (SPSS, version 17.0, SPSS Inc.) or by using GraphPad Prism.

## Results

### *In utero* SHS Exposure Predisposes to Aggravated Elastase-Induced Emphysema in Female Mouse Offspring

**Figure 2:**
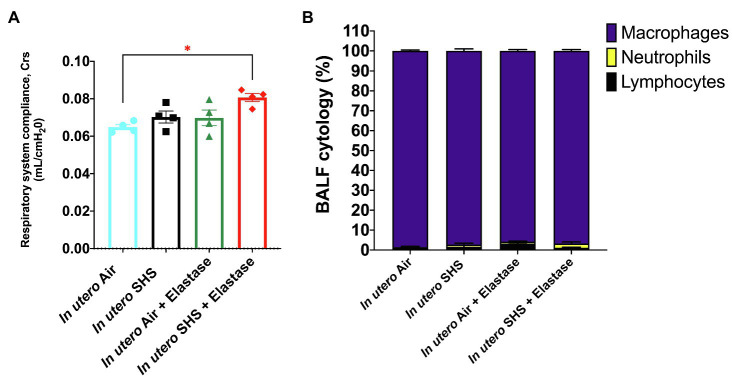
*In utero* SHS plus adult elastase increased the respiratory system compliance without affecting BALF cytology 28days after elastase treatment. **(A)** Respiratory system compliance (Crs) measured using a *FlexiVent* system at 15weeks of age in female mouse offspring. *N*=4 per group; data are expressed as mean±SEM. ANOVA followed by the Tukey’s test for multiple comparisons, ^*^p <0.05: statistically different from *in utero* air group. **(B)** Cytology analysis showing the percentage of leukocytes recovered from BALF of female mouse offspring at 15weeks of age. *N*=5 to 8 per group; data are expressed as mean±SEM.

**Figure 3:**
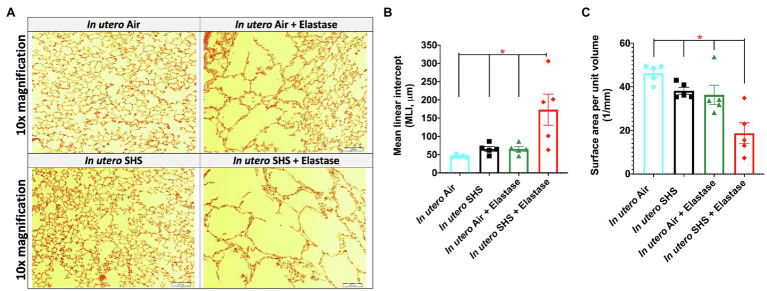
*In utero* SHS plus adult elastase increased mean linear intercept and decreased lung surface area. **(A)** Representative images of H&E stained lung slides from 15-week-old female mouse offspring. **(B)** Quantification of lung tissue enlargement using mean linear intercept (MLI) and **(C)** surface area per unit volume. *N*=5 per group; data are expressed as mean±SEM. ANOVA followed by Tukey’s test for multiple comparisons, ^*^p <0.05: *in utero* SHS+elastase group statistically different from all other groups.

**Figure 4:**
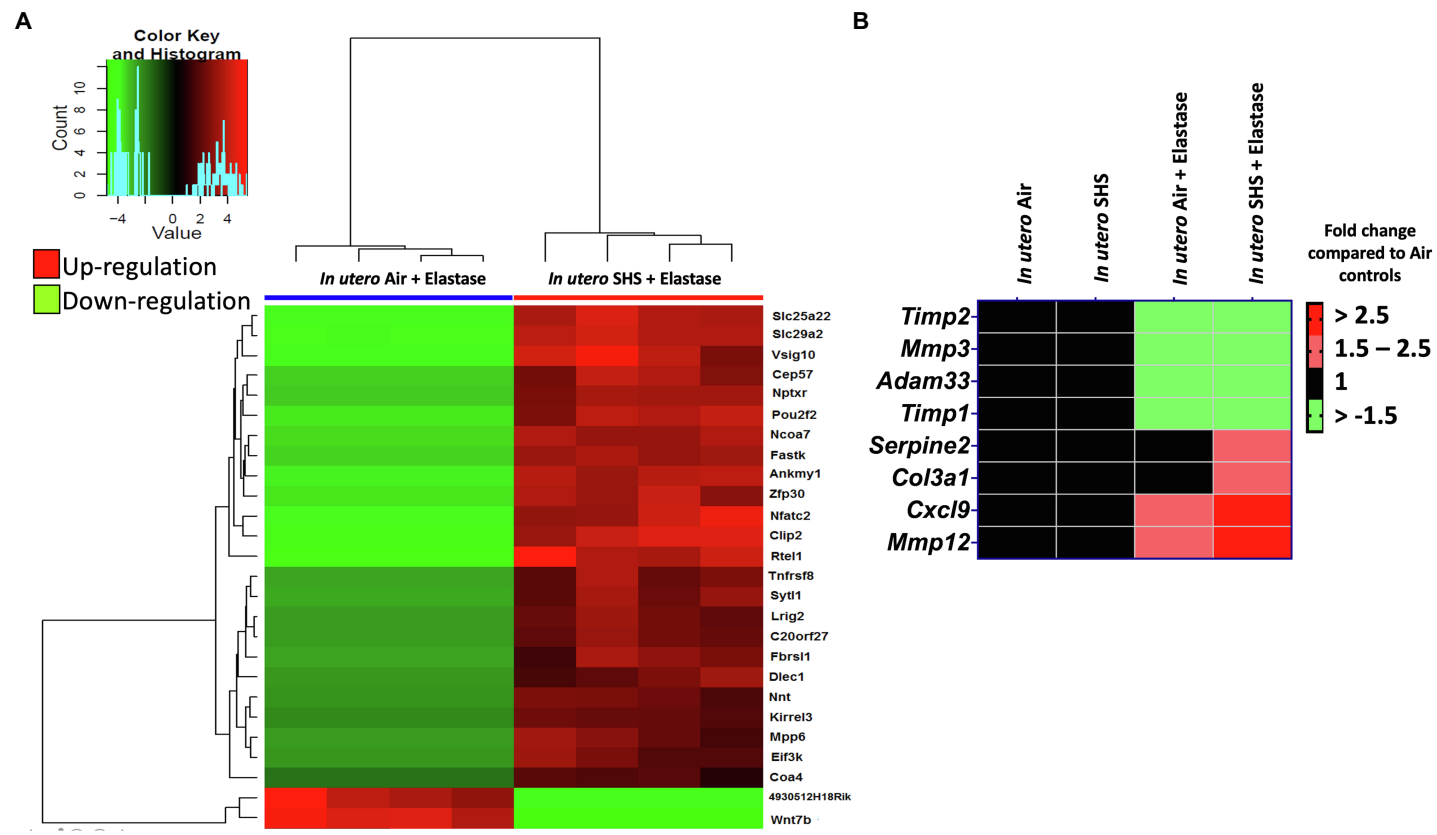
*In utero* SHS plus adult elastase dysregulated lung gene expression. **(A)** Lung RNA sequencing results demonstrate that *in utero* SHS exposure plus elastase up-regulated 24 genes and down-regulated 2 genes when compared to the respective *in utero* air plus elastase control group at 15weeks of age in female mouse offspring. *N*=4 per group. Heatmap displays lung genes with a 2-fold up-/down-regulation (*p*<0.05) and a false discovery rate<0.05. **(B)** Heatmap displays gene expression data obtained by qRT-PCR from the lungs of 15-week-old female mouse offspring. *N*=4–5 per group. Data are expressed as mean per group. Genes displayed have fold-changes > ±1.5 up-/down-regulation compared to the *in utero* air plus saline control group and were considered significant.

Previously we reported (Noël et al., 2017) that pulmonary alveoli were significantly enlarged and that *Serpina1a*, the mouse ortholog of the human gene alpha-1 antitrypsin (α1AT) – a known genetic risk factor for emphysema – was significantly down-regulated in 15-week-old female mouse offspring that had been exposed solely to SHS *in utero*. Those results strongly suggested that *in utero* SHS exposure alone can affect emphysema development in adulthood. Thus, we used an elastase-induced emphysema mouse model here to investigate whether *in utero* SHS exposures can affect subsequent emphysema development in female mouse offspring. Using the flexiVent system, we found that *in utero* SHS plus elastase significantly (*p*<0.05) increased the respiratory system compliance (Crs) in 15-week-old female offspring compared to *in utero* air controls (Figure 2A). This parameter, Crs, assesses the ability of the lungs, the chest walls, and the airways to expand/distend (Vanoirbeek et al., 2010). This effect on lung function, however, was not accompanied by changes in BALF cytology 28days after the elastase treatment (Figure 2B). Further, *in utero* SHS plus elastase significantly (*p*<0.05) increased MLI values (173μm±43; Figure 3B) and decreased lung surface area (18.7 1/mm±4.7; Figure 3C) compared to all other groups, including in the *in utero* air plus elastase group (65.8μm±6.4 and 36.3 1/mm±4.5, respectively). The changes in these two lung structure parameters, MLI and surface area, indicate enlarged airspaces and show that *in utero* SHS exposure predisposes to aggravated alveolar wall destruction in this elastase-induced emphysema model (Figure 3). Moreover, we found that *in utero* SHS plus elastase dysregulated lung gene expression (Figure 4). RNA sequencing of lung tissue revealed up-regulation of 24 genes and down-regulation of 2 genes associated with COPD/emphysema in the *in utero* SHS plus elastase treated mice compared to their respective air controls (Figure 4A). Complementary qPCR analysis additionally showed expression of lung genes associated with protease-antiprotease imbalance, including: metallopeptidase inhibitors, *Timp1* & *2* (-1.5-fold), metallopeptidase domain 33 (*Adam33*; -1.8-fold), serpin peptidase inhibitor clade E member 2 (*Serpine2*; 1.6-fold), as well as matrix metalloproteinase3 (*Mmp3*; -2-fold). Of particular relevance to the present study, *Mmp12* (10.3-fold) was dysregulated following elastase treatment of the offspring exposed *in utero* to SHS (Figure 4B). This suggests that airway remodeling molecular pathways are active in this system. Taken together, these data demonstrate that this elastase-induced emphysema mouse model may mimic human emphysema pathogenesis, in addition to providing insights on how *in utero* SHS exposures contribute to this disease.

### *In utero* Exposure to Low Levels of SHS Predisposes to Aggravated HDM-Induced Asthma in Female Mouse Offspring

**Figure 5:**
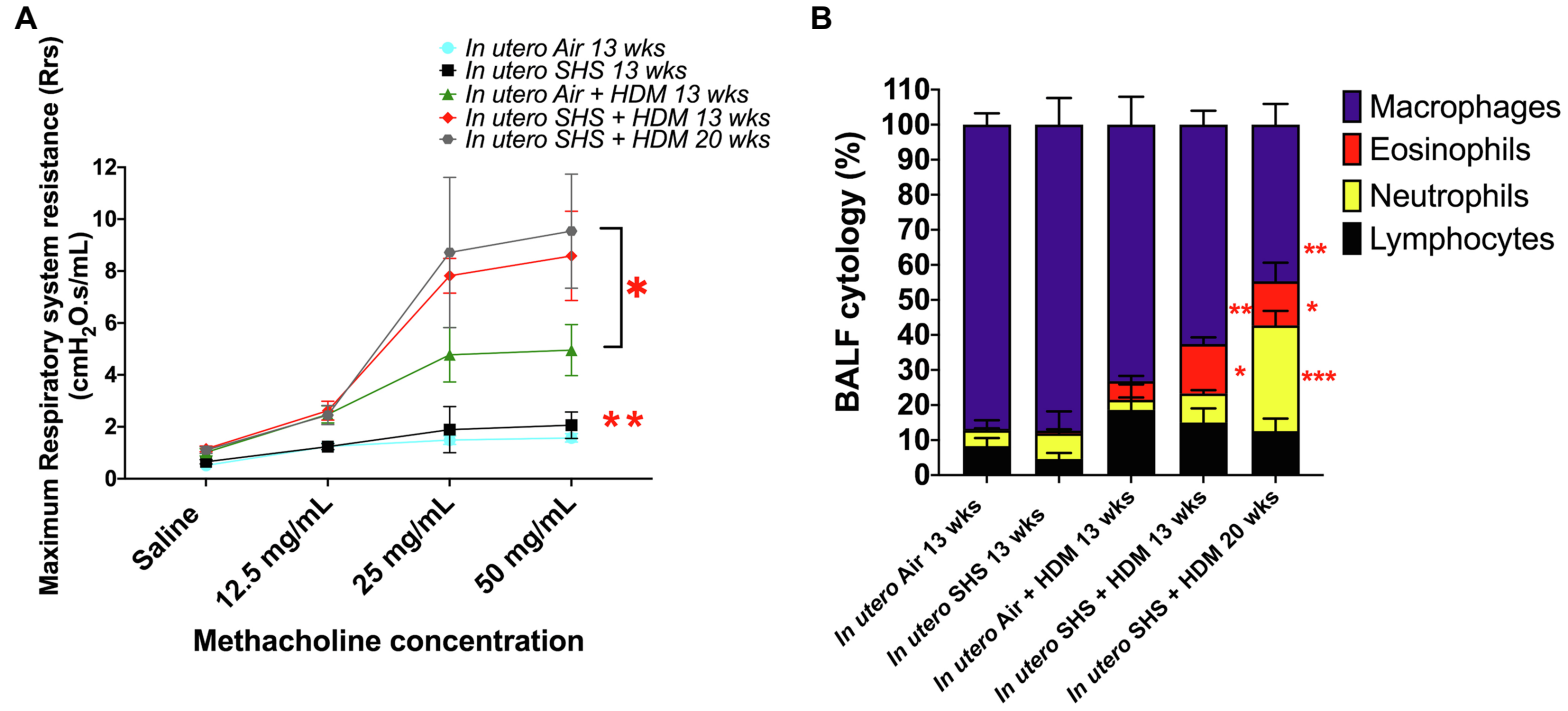
3mg/m^3^ of *in utero* SHS plus adult HDM increased lung resistance and altered BALF cytology. **(A)** Maximum respiratory system resistance (Rrs) measured using a *FlexiVent* system at 13 and 20weeks of age in female mouse offspring. *N*=6–10 per group; data are expressed as mean±SEM. ANOVA followed by Tukey’s test for multiple comparisons, ^*^p <0.05: *in utero* SHS plus HDM group statistically different from *in utero* air plus HDM group, ^**^p <0.05: *in utero* air or SHS plus HDM groups statistically different from *in utero* air or SHS plus saline groups. **(B)** Cytology analysis showing the percentage of leukocytes recovered from BALF of female mouse offspring at 13 and 20weeks of age. *N*=9–10 per group; data are expressed as mean±SEM. ANOVA followed by the Tukey’s test for multiple comparisons, eosinophils: ^*^p <0.05: statistically different from *in utero* air or SHS plus saline and *in utero* air plus HDM groups; macrophages: ^**^p <0.05: statistically different from *in utero* air or SHS plus saline groups; neutrophils: ^***^p <0.05: statistically different from all other groups.

**Figure 6:**
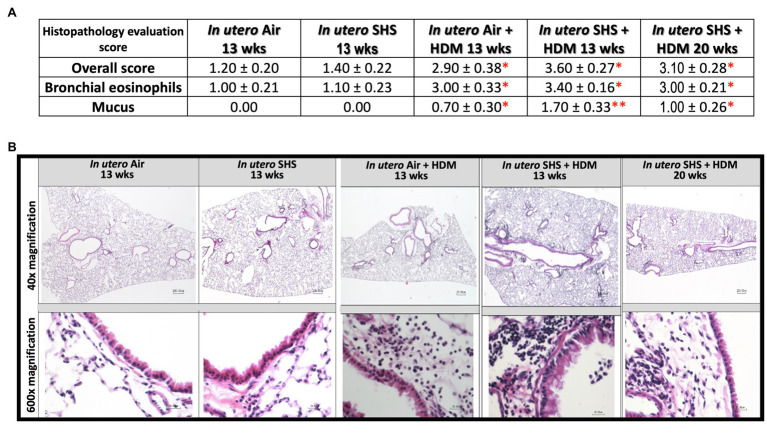
Lung histopathology confirmed inflammation and shows mucus production following HDM treatment. **(A)** Histopathology evaluation score of H&E stained lung tissue of 13- and 20-week-old female mouse offspring. Overall inflammation score was 0=normal, 1=detectable at 100×, 2=obvious at 20× localized, 3=obvious at 20× widespread, 4=complete bronchial cuffs localized, 5=complete bronchial cuffs widespread. All other parameters: 0=normal. 1=minimal, 2=mild, 3=moderate, 4=severe. *N*=9–10 per group, data are expressed as mean±SEM. Kruskal-Wallis test, ^*^p <0.05: statistically different from *in utero* air or SHS plus saline groups; ^**^p <0.05: statistically different from *in utero* air plus HDM group. **(B)** Representative images of H&E stained lung slides from 13 and 20-week-old female mouse offspring.

**Figure 7:**
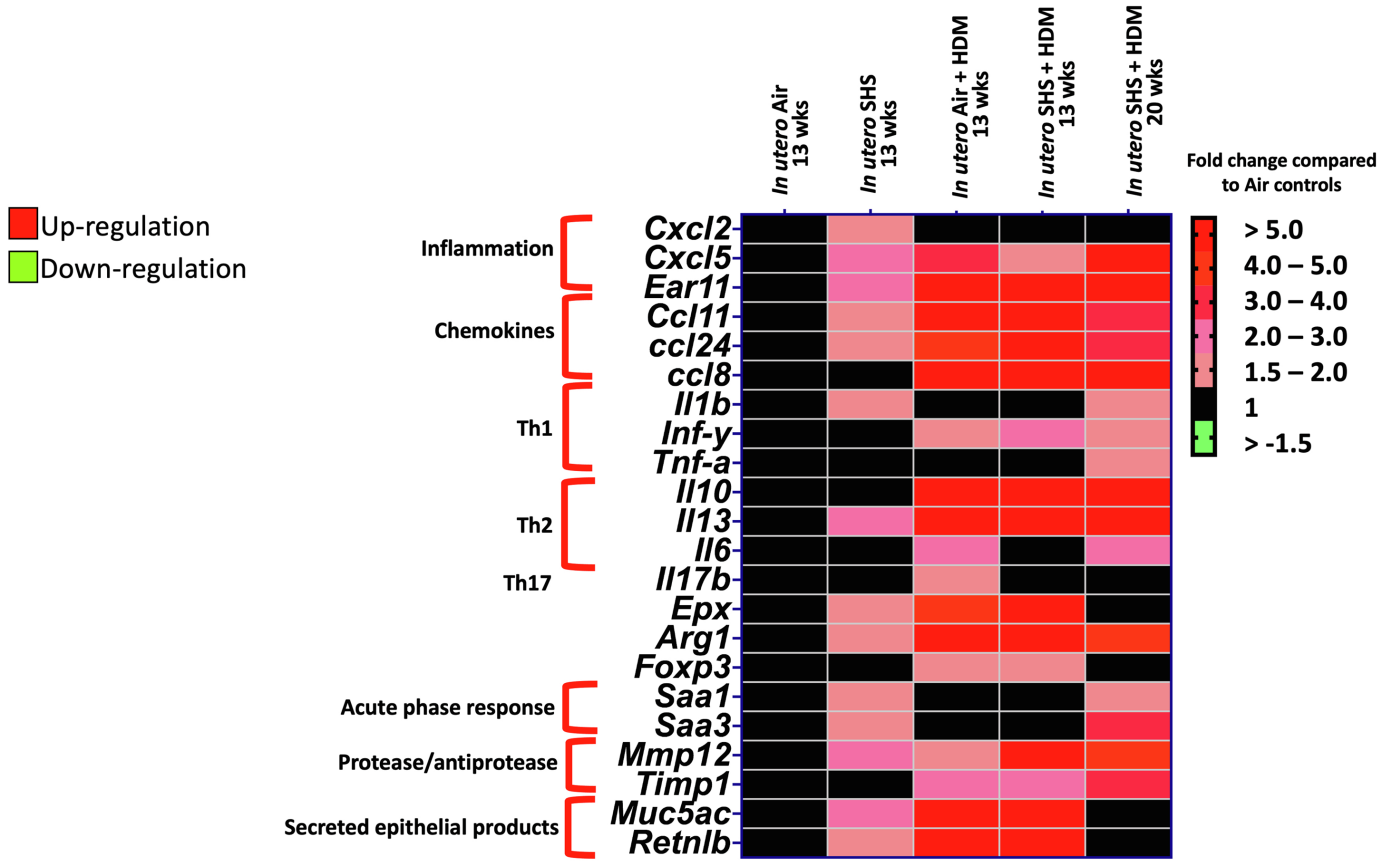
*In utero* SHS plus adult HDM increased the expression of lung genes involved in chemotaxis and activation of eosinophils. Heatmap displays gene expression data obtained by qRT-PCR from the lungs of 13- and 20-week-old female mouse offspring. *N*=9–10 per group. Data are expressed as mean per group. Genes displayed have fold-changes > ±1.5 up-/down-regulation compared to the *in utero* air plus saline control group and were considered significant.

Epidemiological as well as experimental studies have demonstrated that *in utero* SHS exposure is a risk factor for asthma (Stick, 2006; Gibbs et al., 2016; McEvoy and Spindel, 2017). We previously reported that *in utero* exposure to 10mg/m^3^ of SHS aggravates pro-asthmatic responses in ovalbumin-challenged adult male and female mice (Xiao et al., 2013). Here, using an HDM-induced asthma mouse model, we investigated whether a lower concentration of *in utero* SHS, 3mg/m^3^, predisposes to aggravated HDM-induced asthmatic responses at 13 and 20weeks of age in females offspring. We found that 3mg/m^3^ of *in utero* SHS plus adult HDM significantly (*p*<0.05) increased the maximum respiratory system resistance (Rrs) at the methacholine dose of 50mg/ml and altered BALF cytology in both 13- and 20-week-old offspring (Figure 5). This increase in pulmonary resistance (Figure 5A) implies obstruction of airflow in the bronchi, which may be due to the mixed eosinophilic/neutrophilic BALF inflammation that we observed in the 20-week-old offspring and the eosinophilic inflammation noted in the 13-week-old offspring (Figure 5B). Eosinophilic inflammation is a characteristic of asthma and contributes to airflow obstruction. In addition, histopathology results confirmed the BALF inflammation, since we observed significant lung tissue inflammation in the mice exposed *in utero* to SHS and then to HDM as adults (Figure 6). This group also exhibited increased mucus production (Figure 6), a hallmark of asthma exacerbation that may also contribute to airway obstruction (van Rijt et al., 2012; Eyring et al., 2015). Results (Figure 7) at the molecular level support the data obtained at the cellular and tissue levels, with the *in utero* SHS plus HDM treatment increasing expression of genes involved in chemotaxis: Ccl8 (18-fold increase at 13weeks of age compared to 12-fold increase for the *in utero* air + HDM group at 13weeks of age); Ccl24 (6.6-fold increase at 13weeks of age compared to 4.5-fold increase for the *in utero* air + HDM group at 13weeks of age); activation of eosinophils, Epx (11.5-fold increase at 13weeks of age compared to 4.2-fold increase for the *in utero* air + HDM group at 13weeks of age); Th2 inflammation, Il-10 (16.2-fold increase at 13weeks of age compared to 10.3-fold increase for the *in utero* air + HDM group at 13weeks of age), and mucus production, Muc5ac (16.4-fold increase at 13weeks of age compared to 12.7-fold increase for the *in utero* air + HDM group at 13weeks of age). Furthermore, *in utero* SHS exposure plus HDM up-regulated expression of *Mmp12* (5.7-fold increase at 13weeks of age compared to 1.9-fold increase for the *in utero* air + HDM group at 13weeks of age). *Mmp12* plays a role in the extracellular matrix degradation that contributes to airway remodeling in asthma pathogenesis (Xie et al., 2005; Lagente et al., 2009).

### Increased Extracellular Matrix Remodeling Promotes Urethane-Induced Lung Metastases in Female Mouse Offspring Exposed *in utero* to SHS

**Figure 8:**
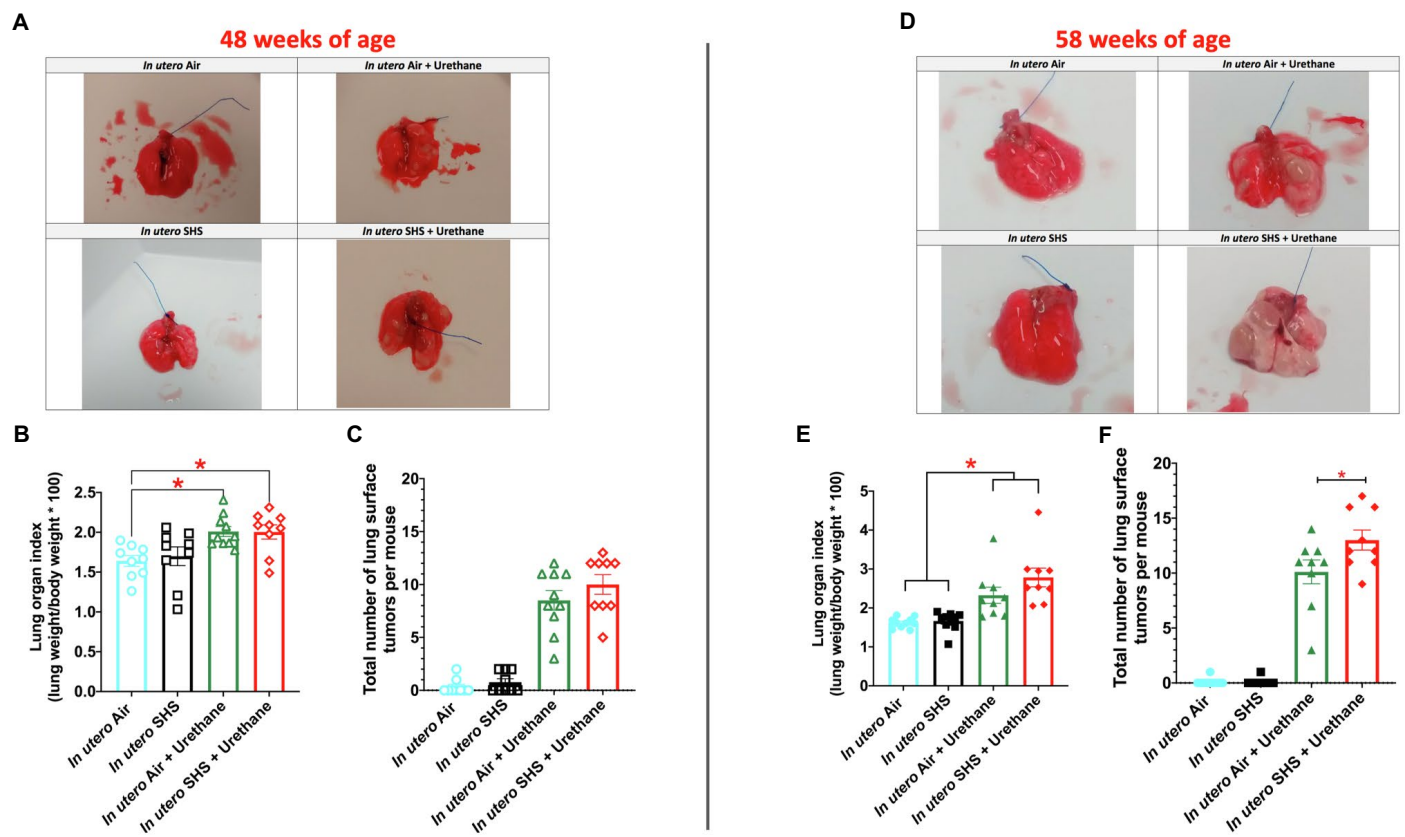
*In utero* SHS plus adult urethane increased the lung organ index and the total number of lung surface tumors at 58weeks of age. **(A,D)** Representative images of excised lungs from 48- and 58-week-old female mouse offspring, respectively, with urethane, treated groups showing lung surface tumors. **(B,E)** Lung organ index calculated as: (lung weight/body weight)×100. *N*=9–10 per group, data are expressed as mean±SEM. ANOVA followed by the Tukey’s test for multiple comparisons, ^*^p <0.05: statistically different from *in utero* air plus saline group. **(C,F)**. The total number of visible lung surface tumors measured using an electronic digital caliper in 48- and 58-week-old mice, respectively. *N*=9–10 per group, data are expressed as mean±SEM. ANOVA followed by the Tukey’s test for multiple comparisons, ^*^p <0.05: statistically different from *in utero* air plus urethane group.

**Figure 9:**
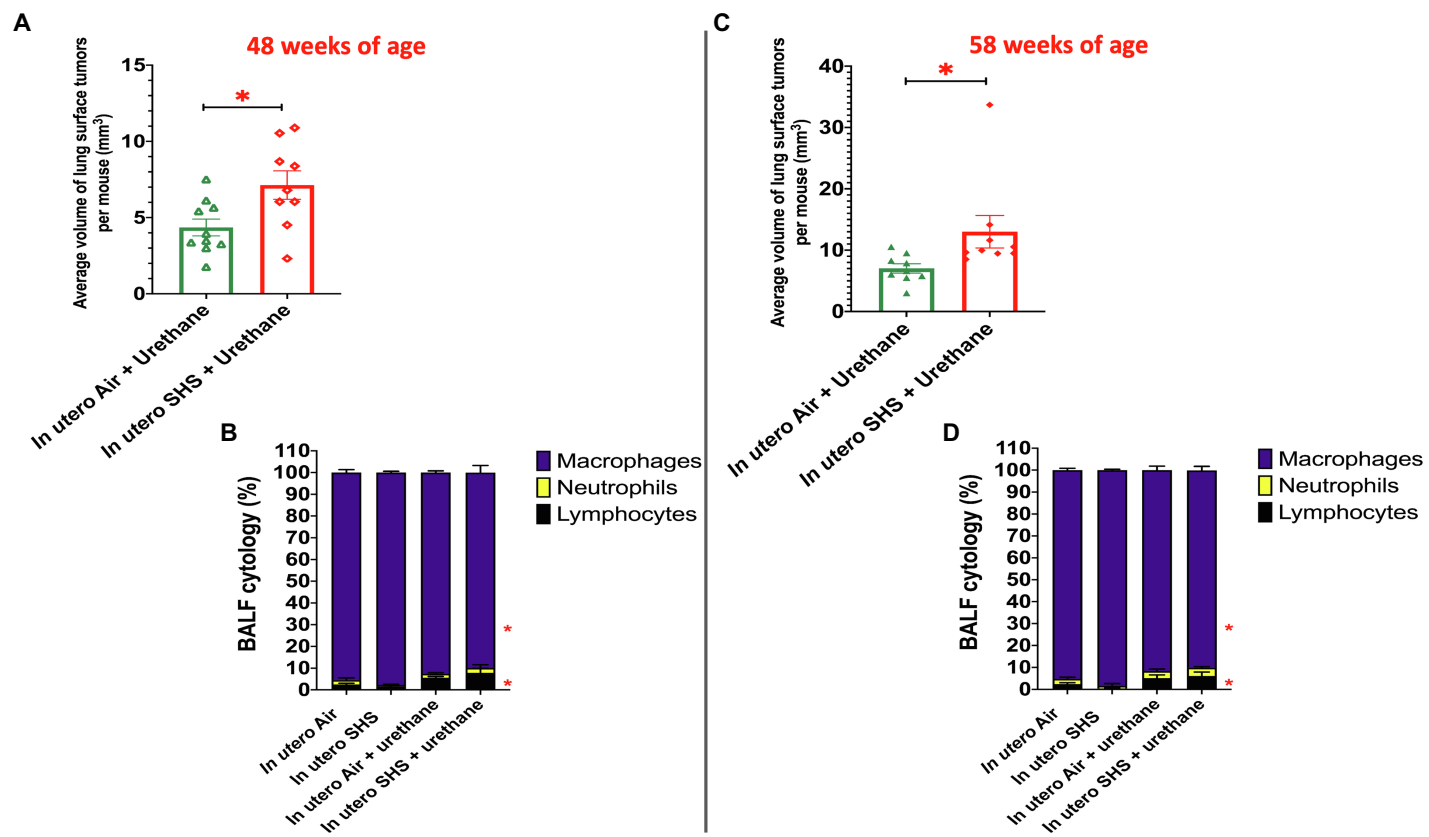
*In utero* SHS plus adult urethane increased the volume of tumors as well as BALF inflammation. **(A,C)** Average volume of lung surface tumors in 48- and 58-week-old female mouse offspring, respectively, treated with urethane as adult. Tumors were estimated to be either spherical or non-spherical. For spherical tumors: volume=4/3 π r^3^; and for non-spherical tumors: volume=0.52 ab^2^ (a=largest and b=smaller diameter). *N*=9–10 per group, data are expressed as mean±SEM. Student *t*-test, ^*^p <0.05: statistically different from *in utero* air plus urethane group. **(B,D)** Cytology analysis showing the percentage of leukocytes recovered from BALF of female mouse offspring at 48 and 58weeks of age, respectively. *N*=9–10 per group; data are expressed as mean±SEM. ANOVA followed by Tukey’s test for multiple comparisons, lymphocytes, and macrophages: ^*^p <0.05: statistically different from *in utero* air plus saline group.

While SHS is a risk factor for lung cancer, it is unclear whether there is an association between *in utero* SHS exposure and adult lung cancer. We previously reported that the lungs of mice exposed *in utero* to SHS and as adults to ovalbumin exhibited up-regulation of miR-155-5p, miR-21-3p, and miR-18a-5p and down-regulation of 16 tumor suppressor genes, all predicted targets of these 3 miRNAs, which have been characterized as oncogenic miRNAs (Xiao et al., 2014). In addition, we showed in 15-week-old mice exposed solely to *in utero* SHS that *Dnmt3a*, which (1) is responsible for *de novo* DNA methylation, (2) plays a key role in epigenetic mechanisms, and (3) whose deficiency in mice is associated with lung cancer, was significantly down-regulated (Noël et al., 2017). Together, these results strongly suggested that *in utero* SHS exposure alone may predispose to adult lung cancer. Next, using a urethane-induced lung cancer mouse model, we investigated whether *in utero* SHS exposures aggravate lung cancer progression in female mouse offspring. No significant body weight differences between groups throughout the study were noted (data not shown). We found that urethane treatment increased the lung organ index at 48 and 58weeks of age (Figures 8B,E). The absence of body weight differences between groups at 48 and 58weeks indicates a significant increase in the weight of the lungs for *in utero* air plus urethane and *in utero* SHS plus urethane, and thus a urethane effect. The total number of lung surface tumors was significantly increased in the *in utero* SHS plus urethane group (13 tumors ±0.9) compared to the respective air controls (10 tumors ±1.1) at 58weeks of age (Figure 8F). Although the total number of lung surface tumors was not significantly different between both urethane groups at 48weeks of age (Figure 8C), the average volume of the surface tumors was significantly higher in the *in utero* SHS plus urethane group (7.1mm^3^±0.9) compared to the respective air controls (4.4mm^3^±0.6; Figure 9A). At 58weeks of age, the difference in the average volume of the surface tumors between the two groups of interest, *in utero* SHS plus urethane (13.0mm^3^±2.6) and *in utero* air plus urethane (7.0mm^3^±0.8) – was even larger (Figure 9C).

**Figure 10:**
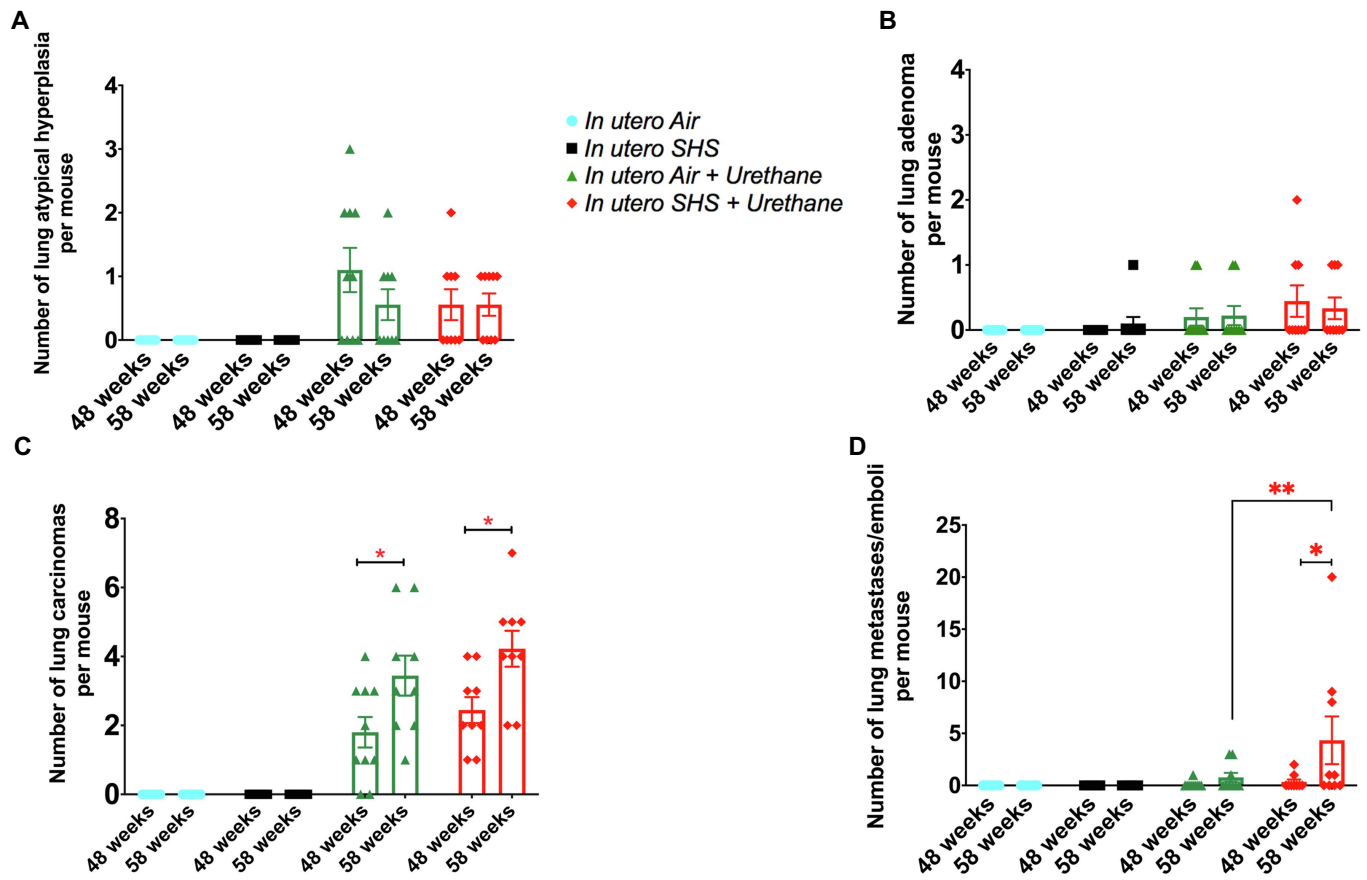
*In utero* SHS plus adult urethane increased the number of intrapulmonary metastases at 58weeks. **(A)** Number of lung atypical hyperplasia observed on H&E stained lung tissue slides. Histopathological evaluation of lungs at 48 and 58weeks of age in female mouse offspring. *N*=9–10 per group, data are expressed as mean±SEM. **(B)** Number of lung adenoma observed H&E stained lung tissue slides. Histopathological evaluation of lungs at 48 and 58weeks of age in female mouse offspring. *N*=9–10 per group, data are expressed as mean±SEM. **(C)** Number of lung carcinoma observed H&E stained lung tissue slides. Histopathological evaluation of lungs at 48 and 58weeks of age in female mouse offspring. *N*=9–10 per group, data are expressed as mean±SEM. Student *t*-test, ^*^p <0.05: statistically different from respective urethane treated group at 48weeks of age (time-course effect). **(D)** Number of lung intrapulmonary metastases observed H&E stained lung tissue slides. Histopathological evaluation of lungs at 48 and 58weeks of age in female mouse offspring. *N*=9–10 per group, data are expressed as mean±SEM. Student *t*-test, ^*^p <0.05: statistically different from respective urethane treated group at 48weeks of age (time-course effect); ^**^p <0.05: statistically different from *in utero* air plus urethane group at 58weeks of age (*in utero* exposure effect).

**Figure 11:**
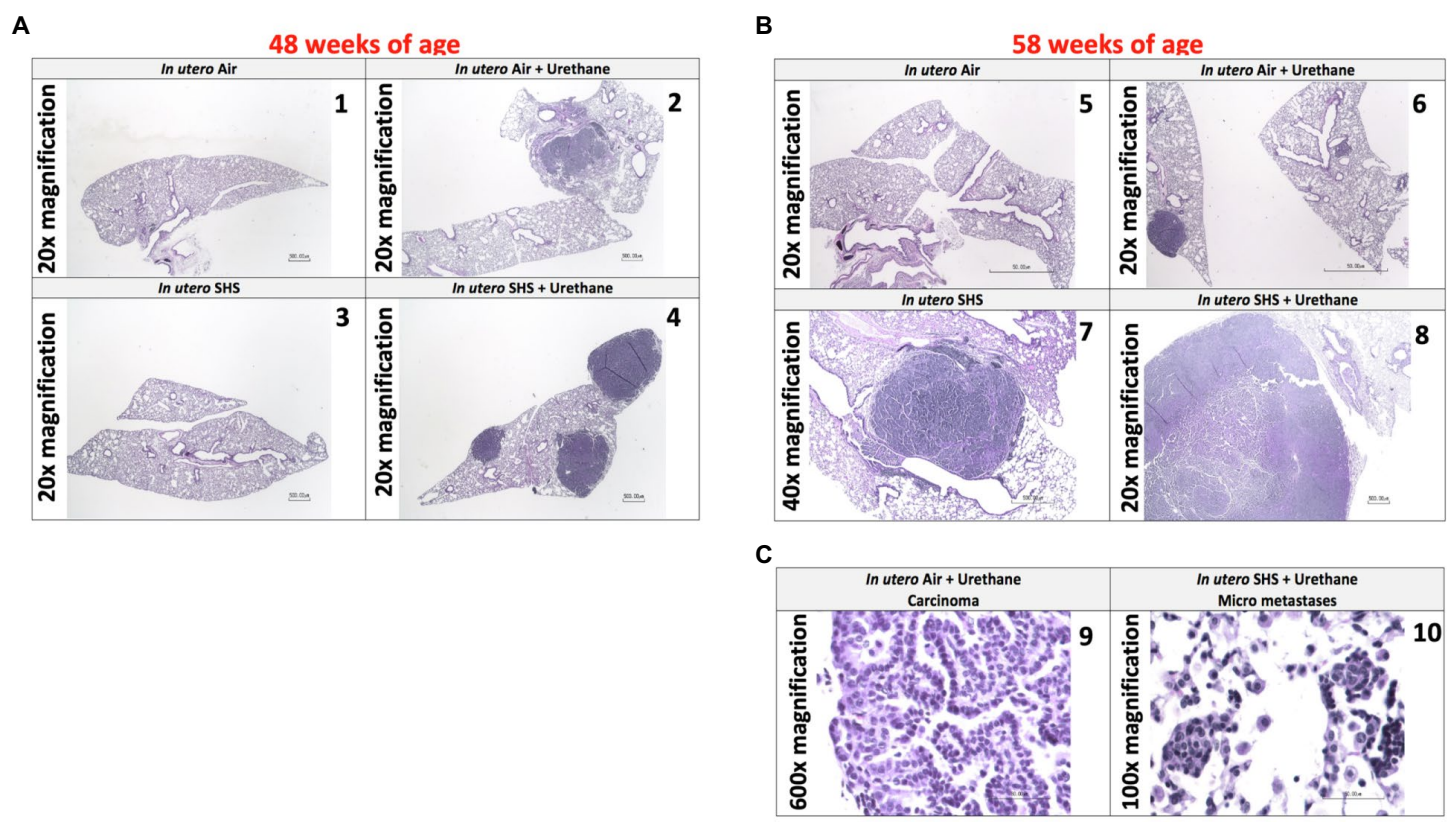
*In utero* SHS plus adult urethane promoted a tumorigenic microenvironment in the lungs- histopathology assessment. **(A)** Representative images of H&E stained lung slides from 48-week-old female mouse offspring; 20× magnification. (1) *In utero* air: normal mouse lung slide; (2) *in utero* air + urethane: mouse lung slide shows 1 carcinoma; (3) *in utero* SHS: normal mouse lung slide; (4) *in utero* SHS+urethane: mouse lung slide shows 3 carcinomas. **(B)** Representative images of H&E stained lung slides from 58-week-old female mice offspring; 20× and 40× magnification. (5) *In utero* air: normal mouse lung slide; (6) *in utero* air + urethane: mouse lung slide shows 1 carcinoma as well as atypical hyperplasia; (7) *in utero* SHS: mouse lung slide shows 1 adenoma; (8) *in utero* SHS+urethane: mouse lung slide shows 1 large carcinoma. **(C)** Representative images of H&E stained lung slides from 58-week-old female mouse offspring. (9) Carcinoma (600× magnification) and (10) micrometastases (100× magnification) are present on the lung slides from *in utero* air + urethane and *in utero* SHS+urethane treated mice, respectively.

**Figure 12:**
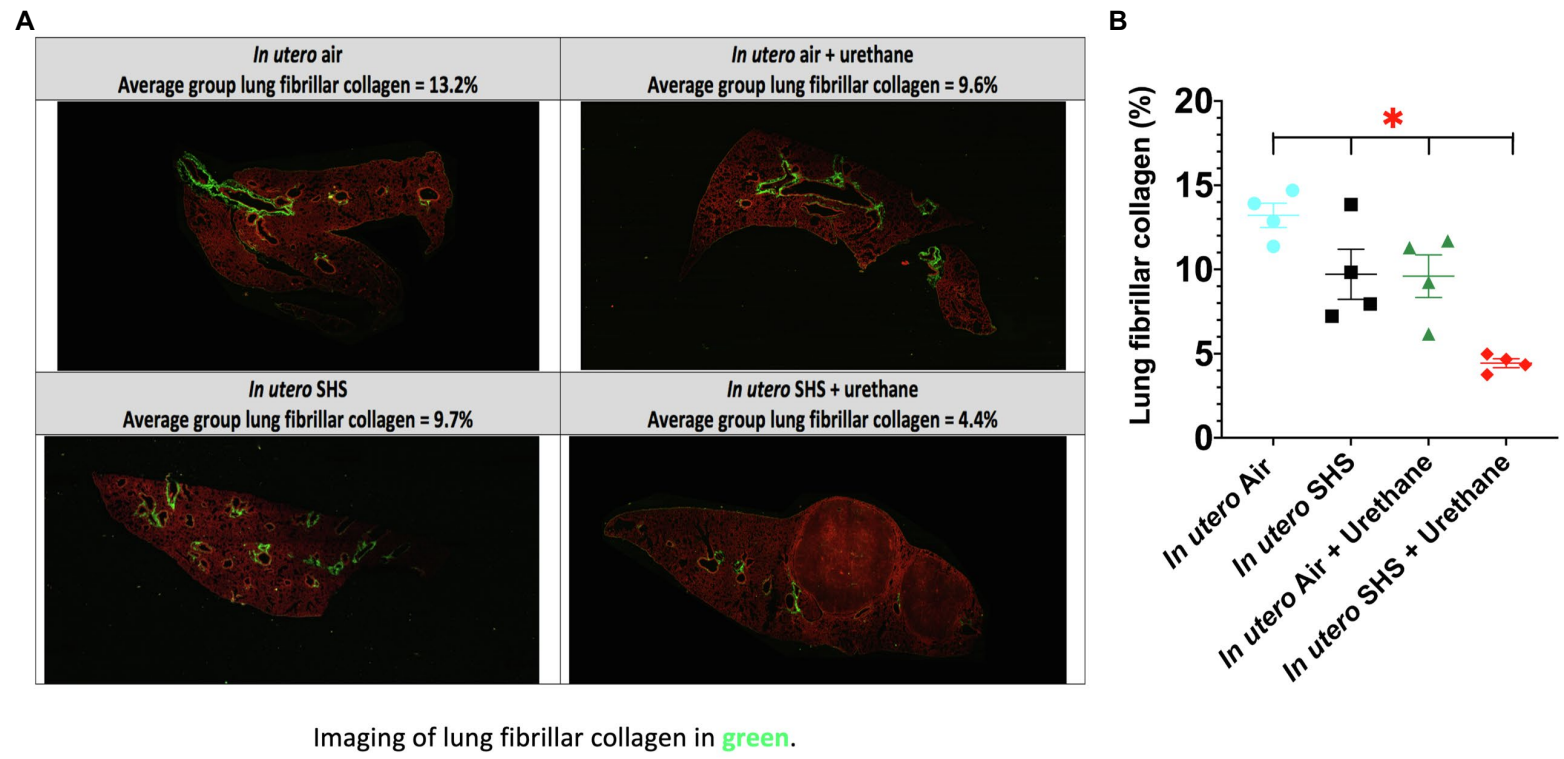
*In utero* SHS plus adult urethane treatment significantly decreased the percentage of lung fibrillar collagen at 58weeks. **(A)** Representative images obtained by second harmonic generation (SHG) microscopy of lungs slides from 58-week-old female mouse offspring. SHG (lung fibrillar collagen) and two-photon excited fluorescence are represented in pseudo color, green and red, respectively. **(B)** Quantification of lung fibrillar collagen content observed by SHG microscopy. For each mouse, three different lung sections were imaged by SHG microscopy. *N*=4 mice per group, data are expressed as mean±SEM. ANOVA, followed by the Tukey’s test for multiple comparisons, ^*^p <0.05: statistically different from all other groups.

**Figure 13:**
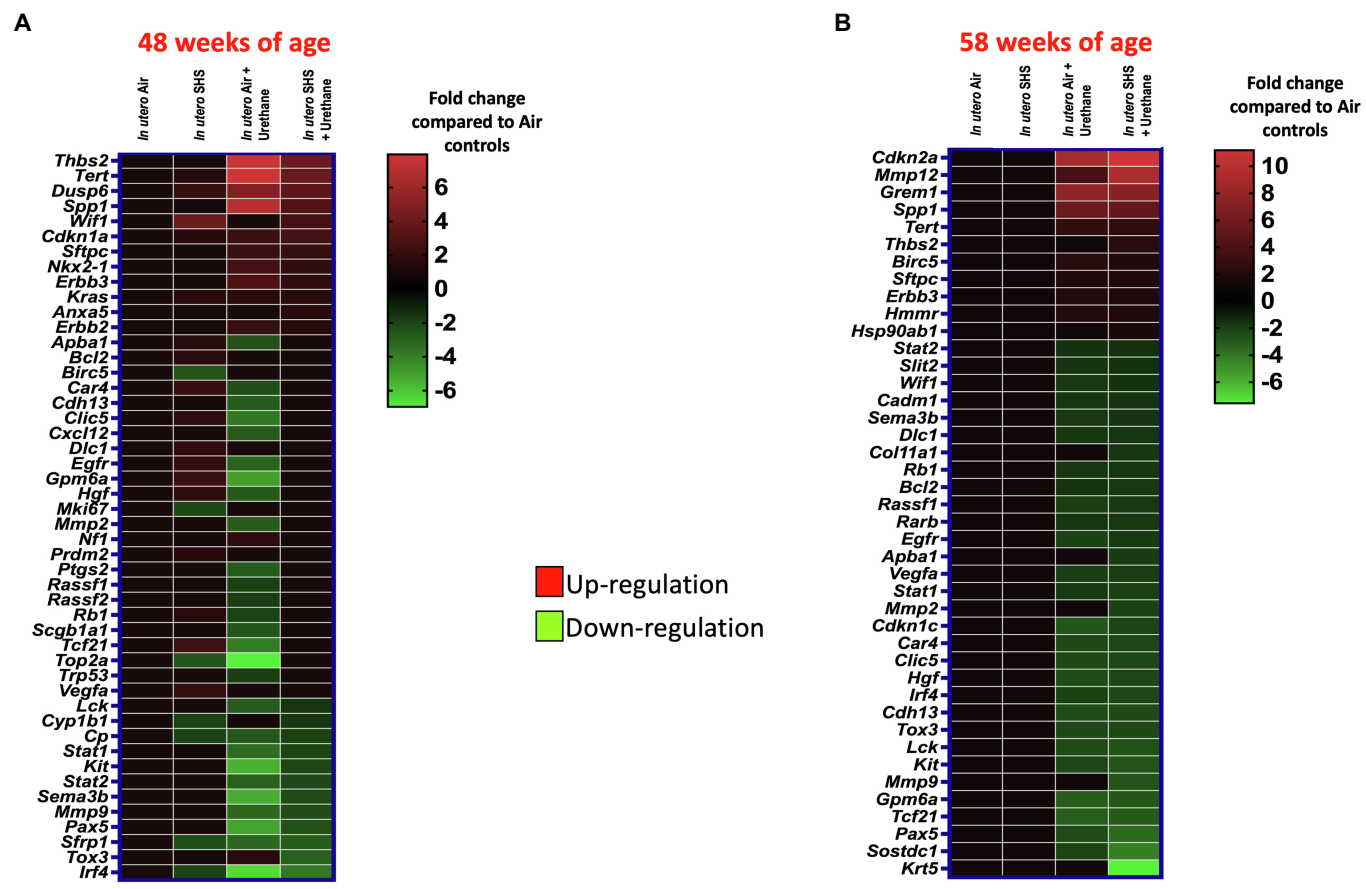
*In utero* SHS plus adult urethane dysregulated the expression of lung genes involved in matrix remodeling and metastasis propagation at 58weeks. **(A,B)** Heatmaps display gene expression data obtained by RT2-PCR array from the lungs of 48- and 58-week-old female mouse offspring, respectively. *N*=4 per group. Data are expressed as mean per group. Genes displayed have fold-changes > ±1.5 up-/down-regulation compared to the *in utero* air plus saline control group and were considered significant.

As for BALF cytology, the *in utero* SHS plus urethane exposures resulted in a chronic lung inflammatory response compared to the *in utero* air plus saline control groups: increased lymphocyte percentage at both 48 (7.9%±2.0 vs. 2.5%±0.5) and 58weeks of age (6.1%±1.8 vs. 2.4%±0.6; Figures 9B,D). These results suggest that *in utero* SHS exposure accelerates tumor proliferation and aggravates BALF inflammation in a urethane-induced mouse model (Figure 9). Histopathological analysis of the lung tissue revealed that BALB/c mice in this urethane-induced lung cancer model exhibited lung lesion changes, including atypical hyperplasia, adenomas, carcinomas, and intrapulmonary metastases (Figures 10, 11). This sequential progression of lung cancer development in mice is similar to the human disease (Kellar et al., 2015). For both groups of mice treated with urethane, the number of lung carcinomas increased from 48 to 58weeks of age (Figure 10C). Furthermore, *in utero* SHS plus urethane mice exhibited increased numbers of intrapulmonary metastases at 58weeks (4.3±2.3) compared to *in utero* air plus urethane mice (0.8±0.4; Figures 10D, 11B,C). Besides, SHG imaging revealed that the *in utero* SHS plus urethane treatment significantly decreased the lung fibrillar collagen content compared to all other groups (Figure 12). The ECM composition of the lungs includes fibrillar collagens, and these proteins contribute significantly to the lung architecture (Burgstaller et al., 2017). Thus, the histopathology assessment and the lung structure results strongly suggest that *in utero* SHS plus urethane promoted a tumorigenic microenvironment in the lungs. At the molecular level, we found that *in utero* SHS plus urethane dysregulated the expression of several genes involved in cancer progression (Figure 13). Specifically, at 48weeks of age, out of the 84 genes analyzed through the lung cancer PCR array, 24 genes were significantly dysregulated (12 up and 12 down) in the *in utero* SHS plus urethane group compared to the air control group (Figure 13A). At 58weeks of age, the *in utero* SHS plus urethane treatment resulted in 42 dysregulated genes (out of the 84 genes from the lung cancer PCR array) compared to the air control groups (Figure 13B). While 11/42 genes were up-regulated, 31/42 genes were down-regulated (Figure 13B). Moreover, several ECM genes, including *Mmp3*, *Mmp9*, *Mmp12*, and collagen type IX alpha 1 chain (*Col11a1*) were dysregulated by the *in utero* SHS plus urethane treatment (Figure 13B). *Mmp12*, which was up-regulated by 9.3-fold in the *in utero* SHS plus urethane group (Figure 13B), has been found to be significantly increased in lung tumors, including NSCLC in humans (Hofmann et al., 2005; Shah et al., 2010; Eide et al., 2016; Ella et al., 2018). Overall, our data show that *in utero* SHS accelerates lung cancer progression in a urethane-induced mouse lung cancer model.

### *In utero* SHS Exposures Increased MMP12 Gene and Protein Expression in Three Distinct Adult Lung Disease Models

**Figure 14:**
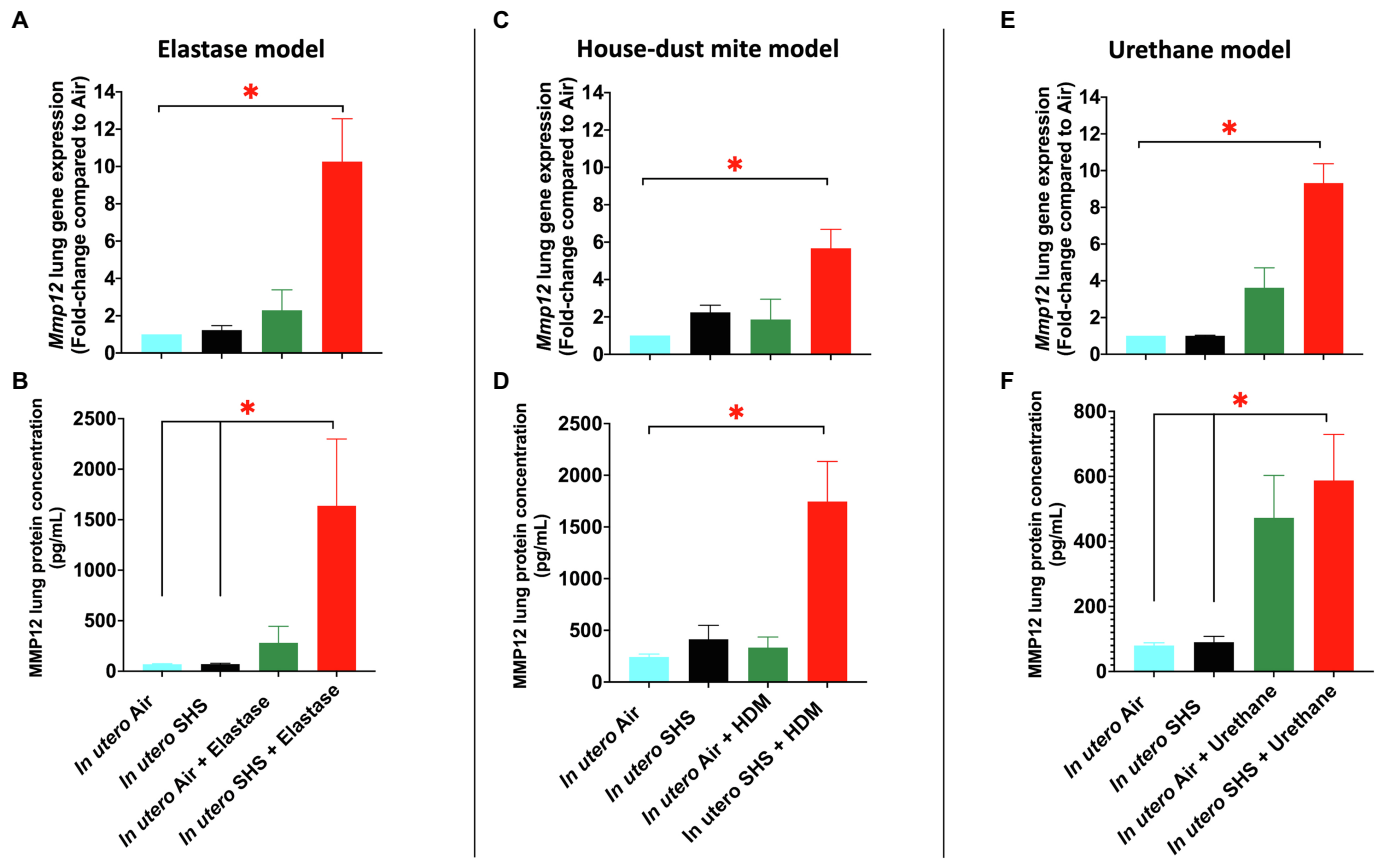
*In utero* SHS exposures increased MMP12 gene and protein expression in three distinct adult lung disease models. **(A)** *Mmp12* lung gene expression results obtained by qPCR in the *in utero* SHS plus elastase-induced emphysema model. *N*=4–5 per group. Data are expressed as mean per group. Gene expression data are displayed as fold-changes compared to the *in utero* air plus saline control group. ^*^p <0.05: statistically different from all other groups. **(B)** MMP12 lung protein expression results obtained by multiplex assay in the *in utero* SHS plus elastase-induced emphysema model. *N*=4 per group. Data are expressed as mean±SEM. ANOVA, followed by the Tukey’s test for multiple comparisons, ^*^p <0.05: statistically significant. **(C)** *Mmp12* lung gene expression results obtained by qPCR in the *in utero* SHS plus HDM-induced asthma model. *N*=9–10 per group. Data are expressed as mean per group. Gene expression data are displayed as fold-changes compared to the *in utero* air plus saline control group. ^*^p <0.05: statistically different from all other groups. **(D)** MMP12 lung protein expression results obtained by multiplex assay in the *in utero* SHS plus HDM-induced asthma model. *N*=4 per group. Data are expressed as mean±SEM. ANOVA, followed by the Tukey’s test for multiple comparisons, ^*^p <0.05: statistically significant from all other groups. **(E)** *Mmp12* lung gene expression results obtained by RT2-PCR array in the *in utero* SHS plus urethane-induced lung cancer model. *N*=4 per group. Data are expressed as mean per group. Gene expression data are displayed as fold-changes compared to the *in utero* air plus saline control group. ^*^p <0.05: statistically different from all other groups. **(F)** MMP12 lung protein expression results obtained by multiplex assay in the *in utero* SHS plus urethane-induced lung cancer model. *N*=4 per group. Data are expressed as mean±SEM. ANOVA, followed by the Tukey’s test for multiple comparisons, ^*^p <0.05: statistically significant.

We have shown that the *Mmp12* gene is critical for triggering the *in utero* SHS-aggravated elastase-induced emphysema (Figures 2–4), HDM-induced asthma (Figures 5–7), and urethane-induced lung cancer (Figures 8–13), with significant up-regulation of this gene in the lungs of the exposed female mouse offspring (Figures 14A,C,E). This gene up-regulation was supported at the protein level with significantly increased expression of MMP12 protein in these three lung disease models (Figures 14B,D,F). Compared to air controls, the *in utero* air plus elastase-treated mice showed up-regulation of *Mmp12* gene by 2.3-fold and of MMP12 protein by 4.1-fold, while the *in utero* SHS plus elastase-treated mice showed up-regulation of the *Mmp12* gene by 10.3-fold and of MMP12 protein by 23.7-fold (Figures 14A,B). In the HDM-induced asthma model, compared to air controls, we found that *in utero* air plus HDM treatment upregulated *Mmp12* gene expression by 1.9-fold and of MMP12 protein by 1.4-fold, while *in utero* SHS plus HDM treatment up-regulated *Mmp12* gene expression by 5.7-fold and of MMP12 protein by 7.2-fold (Figures 14C,D). Finally, compared to air controls, the *in utero* air plus urethane-treated mice showed up-regulation of the *Mmp12* gene by 3.6-fold and of MMP12 protein by 5.9-fold. In contrast, the *in utero* SHS plus urethane-treated mice showed up-regulation of the *Mmp12* gene by 9.3-fold and MMP12 protein by 7.3-fold (Figures 14E,F). Taken together, our data at the gene and protein levels (Figure 14) strongly suggest that MMP12 is central to the development of otherwise unrelated adult lung diseases in mice exposed *in utero* to SHS.

## Discussion

The ‘developmental origins of health and disease’ hypothesis suggests that risk factors for the intrauterine environment can affect fetal development during critical periods, thereby increasing the risk of developing specific diseases later in life (Barker, 1990; Wadhwa et al., 2009; Harding and Maritz, 2012; Carpinello et al., 2018). Numerous epidemiological and experimental studies have associated maternal exposures to outdoor air pollution, CS, and SHS during pregnancy with adverse effects on lung maturation and function in newborns (Swanson et al., 2009; Weldy et al., 2013; Madhloum et al., 2017; Tanwar et al., 2017; Rumrich et al., 2020; Steinle et al., 2020); however, the long-lasting consequences of altered lung structure and function on the onset of chronic lung diseases are poorly documented. The present study investigated the contribution of *in utero* SHS exposures in three distinct models of adult lung diseases: asthma, emphysema, and lung cancer. Compared to air-elastase controls, in the elastase-induced emphysema model, we demonstrated that 15-week-old female mouse offspring exposed *in utero* to SHS had significantly enlarged airspaces plus upregulated expression of *Mmp12* (10.3-fold; Figures 2–4). Compared to air HDM controls, in the HDM-induced asthma model, at 13weeks of age, *in utero* exposures to SHS increased the percentage of eosinophils in BALF and potentiated *Mmp12* gene expression (5.7-fold; Figures 5–7). Furthermore, compared to air urethane controls, in the urethane-induced lung cancer model, *in utero* exposures to SHS significantly increased the volume of the tumors, as well as the number of intrapulmonary metastases, and up-regulated *Mmp12* (9.3-fold) in 58-week-old female mouse offspring (Figures 8–13). Overall, our data in three different mouse models of human lung diseases show that *in utero* SHS exposures aggravated the severity of elastase-induced emphysema, HDM-induced asthma, and urethane-induced lung cancer (Figures 2–13). The common mechanisms by which *in utero* SHS affects these distinct lung diseases are unknown. Our findings, however, revealed that *Mmp12* is upregulated at both the gene and protein levels in these three separate adult lung disease models following *in utero* SHS exposures (Figure 14). Thus, our data point to MMP12 as a critical protein involved in *in utero* SHS-aggravated lung tissue destruction and remodeling (Figures 3A, 4, 6B, 7, 11–14). Hence, in our highly controlled *in utero* SHS exposure studies, we showed that MMP12 is up-regulated by *in utero* SHS and is a key factor predisposing to aggravated adult lung responses (Figure 14). Taken together, our study, for the first time, indicates that *in utero* SHS exposures aggravated subsequent adult outcomes in three otherwise unrelated, severe, and often fatal lung diseases.

CS exposures lead to continuous activation of lung macrophages (Xu et al., 2017). Alveolar macrophages produce *Mmp12*, which is critical for lung structural remodeling in CS-associated lung diseases, including emphysema, asthma, and lung cancer. *Mmp12* dysregulation is associated with all three of these diseases (Su et al., 2006; Mukhopadhyay et al., 2010; Xu et al., 2017; Shibata et al., 2018). These effects have been demonstrated following direct exposures to mainstream CS. In contrast, our data in adult mice exposed *in utero* to SHS, which is orders of magnitude lower than CS in terms of exposure levels, showed that *in utero* SHS exposures upregulate the adult lung gene and protein expression of MMP12 in the offspring, associated with the onset of emphysema, asthma, and lung cancer (Figures 2–14). Our results suggest that similarly to adult CS exposures, *in utero* SHS exposures affect MMP12 gene and protein regulation. Since *in utero* exposures to SHS do not involve direct exposures of the developing lungs to this toxicant, other mechanism(s) must be operating. Prenatal exposures to environmental pollutants, including arsenic, air pollution, phthalates, and tobacco smoke, during susceptible windows of fetal development, dysregulate the epigenome and increase the risk for adult cardiovascular diseases, diabetes, asthma and osteoporosis (Perera and Herbstman, 2011; Li et al., 2017; Rauschert et al., 2019). Specifically, *in utero* exposures to CS or SHS alter gene methylation, indicating the involvement of epigenetic mechanisms, which can affect pre- and post-natal lung development (Joubert et al., 2016; Christensen et al., 2017; Meyer et al., 2017). In a cohort study, maternal smoking imprinted lasting altered methylation effects as observed *via* blood DNA methylation status of offspring at birth, as well as at 7 and 17years of age (Richmond et al., 2015). This clearly shows that in humans, *in utero* exposures to CS affect the offspring epigenome, an effect that persists for nearly two decades after exposure. Since MMPs are involved in both lung health and disease, they are highly regulated at many levels, including gene transcription, translation, secretion, proenzyme activation, and endogenous inhibition (Belvisi and Bottomley, 2003; Elkington and Friedland, 2006; Fanjul-Fernández et al., 2010). In humans, *Mmp12* is located on chromosome 11 and is clustered in the 11q22.3 region (Chernov and Strongin, 2011). Epigenetic profiling data of the 11q22.3 MMP cluster revealed that hypomethylation is associated with actively transcribed MMPs (Chernov et al., 2010; Chernov and Strongin, 2011). We previously demonstrated that *in utero* SHS exposures alone result in down-regulated gene and protein expression of DNA methyltransferase 3 alpha (DNMT3A) in adult mouse offspring by 10- and 2.7-fold, respectively (Noël et al., 2017). DNMT3A down-regulation by *in utero* SHS exposures suggests hypomethylation in adult lungs. Since we showed that *in utero* SHS exposures alone can result in hypomethylated offspring lungs (Noël et al., 2017) and earlier studies showed that hypomethylation is linked to effective transcription of MMPs (Chernov et al., 2010; Chernov and Strongin, 2011), it is likely that *in utero* SHS exposures affect *Mmp12* gene regulation *via* epigenetic mechanisms. These could affect fetal lung programming and subsequent lung development. Together, our previously published and current data suggest that epigenetic cues may play an important role in regulating MMP12 activity following *in utero* SHS exposures (Noël et al., 2017; Figure 14). Since *Mmp12* production is induced by CS (Mukhopadhyay et al., 2010; Xu et al., 2017), we hypothesize that *in utero* SHS exposures, *via* epigenetic mechanisms, up-regulate the expression of *Mmp12* after exposure to a second environmental risk factor (‘hit’). Although the exact mechanisms underlying how *in utero* SHS exposures affect *Mmp12* epigenetic regulation remain to be clarified, the epigenetic-mediated regulatory mechanism governing *Mmp12* transcription following *in utero* SHS exposures should provide new insights into the aggravated lung responses following exposure to a second toxicant during adulthood. More research is needed to validate whether hypomethylated *Mmp12* could serve as a potential biomarker for increased susceptibility to adult emphysema, asthma, and lung cancer following *in utero* SHS exposures.

### Emphysema

Chronic obstructive pulmonary diseases and lung cancer are, respectively, the third and sixth leading causes of death worldwide (Viniol and Vogelmeier, 2018); however, the associations between *in utero* SHS exposure and development or exacerbation of COPD and lung cancer remain unclear. Epidemiological evidence has shown that *in utero* or early life exposures to CS and SHS can predispose to emphysema development (Lovasi et al., 2010; Foreman et al., 2011; Kachroo et al., 2020); thus expanding the documented contribution of *in utero* CS/SHS exposures to lung diseases beyond asthma. Emphysema is a progressive, irreversible obstructive lung disease characterized by airspace enlargement (Sharafkhaneh et al., 2008; Ishii et al., 2014). Here, we found a significant widening of airspaces in the group that was exposed *in utero* to SHS and that received elastase as adults, compared to the *in utero* air plus elastase treated counterparts (Figures 3A,B). These lung structural effects, in addition to being consistent with what is observed in human emphysema (Verbeken et al., 1992), suggest that *in utero* SHS exposures of mouse embryos primed the subsequent elastase-induced lung tissue destruction in the adult offspring. This irreversible parenchymal damage translates into functional changes, including increased respiratory system compliance (Figure 2A), which is associated with lower elastic recoil (Comberiati et al., 2020). The differential pressure between boundaries of an elastic structure is labelled recoil pressure. In the lungs, this is the pressure difference between the alveolar and the pleural spaces (Comberiati et al., 2020). Verbeken et al. (1992) demonstrated that in excised emphysematous lungs, there is an association between lung recoil pressure at 80% of total lung capacity and MLI values: as MLI increases, recoil pressure decreases. That study also suggested that increased airspaces are key players leading to altered lung elasticity. It is therefore very likely that the morphological changes that we observed in the lung tissue, as evidenced by increased MLI values for the *in utero* SHS plus elastase group (Figure 3A), was the cause of the lung physiological dysfunction revealed by the increased lung compliance (Figure 2A), corresponding to reduced elastic recoil in that group (Comberiati et al., 2020). Respiratory compliance is critical for normal respiratory function. Increased lung compliance makes breathing more difficult and is associated with obstructive lung diseases, including emphysema (Verbeken et al., 1992).

In addition to lung structural effects, RNA-sequencing revealed that the lung genes dysregulated by the *in utero* SHS plus elastase treatment (Figure 4A) were consistent with emphysema pathogenesis (Kneidinger et al., 2011). The up-regulated genes included: centrosomal protein 57 (*Cep57*; 6.3-fold), a gene identified as part of a network module associating *in utero* SHS exposures with adult COPD in humans (Kachroo et al., 2020); nuclear receptor coactivator 7 (*Ncoa7*; 7.0-fold), whose expression was dysregulated in COPD lung tissue compared to non-disease tissue (Cho et al., 2015); nuclear factor of activated T cells 2 (*Nfatc2*; 7.9-fold), a transcription factor involved in T cell cytokine production, whose dysregulation has been associated with the pathogenesis of COPD exacerbation (Singh et al., 2014); regulator of telomere elongation helicase 1 (*Rtel1*; 8.6-fold), for which it was demonstrated that a single-nucleotide polymorphism in this gene was associated with COPD in a case-control study (Ding et al., 2017); and leucine rich repeats and immunoglobulin like domain 2 (*Lrig2*; 4.8-fold), for which a single-nucleotide polymorphism near this gene was associated with alterations in adjusted density of emphysematous lungs in a genome-wide association study (Kim et al., 2019a; Figure 4A). We also found (Figure 4A) that the gene expression of wingless-type MMTV integration site family member 7B (*Wnt7b*) was down-regulated (-9.2-fold) in the *in utero* SHS plus adult elastase-treated female mice compared to the *in utero* air plus adult elastase group. The results agree with the previously demonstrated research in human and animal studies investigating emphysema pathogenesis (Kneidinger et al., 2011).

Lung connective tissue is composed of elastic fibers, including elastin (Sharafkhaneh et al., 2008). Increased levels of MMP12, which degrades elastin, are central to emphysema pathogenesis both (1) *in vivo*, in MMP12 knockout and overexpressed mouse models exposed to CS (Hautamaki et al., 1997; Morris et al., 2003; Shapiro et al., 2003), and (2) in clinical studies, from analyses of emphysema patients’ sputum, blood and lung tissue samples (Molet et al., 2005; Demedts et al., 2006; Elkington and Friedland, 2006; Chaudhuri et al., 2012; Ishii et al., 2014). We observed a 10.3-fold up-regulation of *Mmp12* gene expression in the *in utero* SHS plus elastase group (Figure 4B). Overexpression of MMP12, involved in airway remodeling, leads to the destruction of alveolar walls during emphysema development (Hautamaki et al., 1997; Morse and Rosas, 2014; Trojanek et al., 2014; Shibata et al., 2018). This significant increase in expression of this protein-coding gene is consistent with the degradation of elastin and the loss of alveolar walls that led to elevated MLI values and increased lung compliance (Figures 2–4). These results indicate that *Mmp12* may be a critical ‘player’ in the destruction of lung tissue in the *in utero* SHS-exposed mice (Figures 3, 4). Enhanced *Mmp12* expression also was supported by the 1.5-fold down-regulation of *Timp1* (Figure 4B). *Timp1* inhibits the activity of *Mmp12*. Thus up-regulation of *Mmp12* and down-regulation of *Timp1* result in a protease-antiprotease imbalance, a hallmark mechanism underlying emphysema progression (Sharafkhaneh et al., 2008). Overall, our findings add to the pool of both experimental and human studies highlighting the importance of heightened MMP12 activity to the development of emphysematous pathological processes (Hautamaki et al., 1997; Churg et al., 2003; da Hora et al., 2005; Molet et al., 2005; Demedts et al., 2006; Xu et al., 2007; Chaudhuri et al., 2012; Ishii et al., 2014). Our study is unique in emphasizing the association between *in utero* SHS exposures and aggravation of adult emphysema, and is the first to show that *Mmp12* is a key element in the *in utero* SHS exposure-induced predisposition to emphysema, following adult exposure to a second toxicant, even though, in our study, there was no direct exposure of the fetal lung to CS or SHS.

### Asthma

*In utero* SHS exposure aggravates ovalbumin-induced asthma by altering lung structure, function, and dysregulating gene expression in adult mice (Penn et al., 2007; Rouse et al., 2007; Xiao et al., 2013). Although those studies highlighted the persistent effects of *in utero* SHS exposures on lung immune responses, the role of *in utero* SHS exposure in asthma pathogenesis is poorly understood. Here, we used HDM allergens which are highly relevant to human allergic diseases, including asthma, as these ubiquitous indoor allergens induce responses from the innate immune system (Yasuda et al., 2020). A previous study showed that *in utero* exposures of C57BL/6 mice to 50mg/m^3^ of cigarette smoke led to exacerbated HDM-induced asthma in terms of increases in airway resistance, eosinophilic inflammation, and Il-4, Il-5, and IgE levels when compared to filtered-air controls (Eyring et al., 2015). Using this physiological HDM-induced asthma mouse model, we were able – in the *in utero* SHS exposed groups – to recapitulate eosinophilic inflammation, at the molecular (Figure 7), cellular (Figure 5B), and tissue (Figure 6) levels, with increased airway resistance (Figure 5A) and airway remodeling (Figures 6, 7). Also, hallmark genes of classical Th2 responses were expressed with a higher fold-change in the *in utero* SHS plus HDM groups compared to the air HDM controls (Figure 7). In addition to BALF cellular infiltrates (Figure 5B), excess mucous in the lung tissue and increased expression of *Muc5ac*, which is involved in mucous production, were observed in the HDM-treated mice (Figures 6, 7). Together, these can result in airway obstruction and contribute to airway dysfunction (Figure 5A).

In severe asthma, the involvement of altered lung function in airway remodeling remains elusive. Airway remodeling is concurrent with eosinophilia, affects lung structure, and involves changes in lung extracellular matrix proteoglycans and goblet cell hyperplasia (Lloyd and Robinson, 2007). In the present study, we found that the gene expression of *Mmp12*, an essential protein-coding gene involved in extracellular matrix remodeling (Xu et al., 2017), was increased by 5.7-fold in the *in utero* SHS plus HDM group, while it was increased by 1.9-fold in the *in utero* air plus HDM group when both were compared to the air plus saline group (Figure 7). In addition, gene expression of *Muc5ac*, a marker of goblet cell hyperplasia (Kim et al., 2019b), was increased by 16.4-fold in the *in utero* SHS plus HDM group, while it was increased by 12.7-fold in the *in utero* air + HDM group when compared to the air plus saline group (Figure 7). In our model, these data suggest allergen-induced airway remodeling later in life, evidenced by increased gene expression of *Mmp12* and *Muc5ac*, which may be primed by *in utero* SHS exposures. Prior studies in cockroach and ovalbumin allergen asthma models revealed that MMP12 is a crucial pro-inflammatory response contributor, especially regarding eosinophil recruitment, that can impact structural remodeling allergic airway diseases (Pouladi et al., 2004; Warner et al., 2004).

Moreover, a case-control study revealed that polymorphism in the *Mmp12* gene predisposes to bronchial asthma (Tacheva et al., 2018). Also in rats, the MMP12 gene and protein expression were up-regulated in an allergic bronchial asthma model (Chiba et al., 2007). These findings have clinical relevance, since an earlier study revealed that the expression of MMP12 was significantly increased in airway smooth muscle of large airways of patients with fatal asthma compared to controls (Araujo et al., 2008). MMP12 gene polymorphism is also considered a risk factor for the development of severe asthma in youth (Mukhopadhyay et al., 2010). Thus, it is plausible that the *in utero* SHS exposure-induced increased expression of *Mmp12* may contribute to the development of severe asthma. Overall, the increased severity of allergic airway diseases by *in utero* exposures to tobacco smoke in rodents is supported by multiple studies (Singh et al., 2003, 2009; Rouse et al., 2007; Blacquiere et al., 2009; Xiao et al., 2013; Eyring et al., 2015). Our results show that *in utero* SHS, at least in this mouse model of asthma, promotes asthma phenotype responses. Our data also show that *in utero* exposure to low levels of SHS exacerbates subsequent impaired lung function and BALF inflammation in an HDM-induced asthma mouse model, similar to results of exposures to 10mg/m^3^ of SHS followed by ovalbumin challenges, as we have previously reported (Xiao et al., 2013). This indicates that there may be no safe levels of SHS exposures during pregnancy regarding asthma development and disease severity in the offspring. Taken together, the present study emphasizes (1) that even *in utero* exposures to low levels of SHS (3mg/m^3^) can potentially lead to the development of serious asthma symptoms in adulthood (Figures 5–7), and (2) that targeting the regulation of MMP12 could be a potential therapeutic approach related to *in utero* SHS exposure-induced enhancement of asthmatic responses.

### Lung Cancer

Lung cancer has a poor prognosis and is responsible for 30% of all cancer-related deaths (Kwon and Berns, 2013). Small cell lung cancer and NSCLC represent approximately 20 and 80% of all lung cancer types, respectively (Kwon and Berns, 2013). NSCLC comprises many different histological presentations, including squamous cell carcinoma and adenocarcinoma (Liu et al., 2016). Squamous cell carcinoma predominately affects smokers, while adenocarcinoma, is mostly observed in non-smokers (Dubin and Griffin, 2020). Urethane, a Group 2A carcinogen, is used extensively to model multistage human lung carcinogenesis (Kwon and Berns, 2013). This lung cancer model allows tumors to develop in mice according to 4 stages: tumor initiation, promotion, malignant conversion, and tumor progression (Kwon and Berns, 2013). In terms of lung pathology, this translates into atypical lung hyperplasia, adenoma, adenocarcinoma, and metastasis (Jackson et al., 2001). Although human and mouse lung tumors are histologically distinct (Nikitin et al., 2004; Pandiri, 2015), in our model, the histological presentation (Figures 10, 11) and the molecular signatures imprinted on the lungs by the urethane treatment – dysregulation of *Kras*, *Anxa5*, *Cdkn2a*, *Sftpc*, *Pax5*, *Sostdc1*, *Krt5* – (Figure 13) suggest the development of NSCLC adenocarcinomas (Kwon and Berns, 2013; Sunaga et al., 2013; Ren et al., 2015; Liu et al., 2016; Sun et al., 2016; Xiao et al., 2017; Zhang et al., 2018). Indeed, at 48weeks of age, the Kirsten rat sarcoma oncogene (*Kras*) was up-regulated by 1.6-fold in the three treatment groups: *in utero* SHS plus saline, *in utero* air plus urethane, and *in utero* SHS plus urethane (Figure 13A). The *Kras* gene is well-known for its oncogenic properties and its critical role in tumorigenesis, including in non-small cell lung cancer (NSCLC; Sunaga et al., 2013). At this same time point, the Annexin A5 (*Anxa5*) gene was up-regulated (1.5-fold) in the *in utero* SHS plus urethane group (Figure 13A). *Anxa5* plays a key role in lung cancer pathogenesis by regulating crucial signaling pathways associated with NSCLC (Sun et al., 2016). At 58weeks of age, up-regulated genes included cyclin-dependent kinase inhibitor 2A (*Cdkn2a*; 11.2-fold) and surfactant protein C (*Sftpc*; 1.9-fold) when compared to the *in utero* air plus saline group (Figure 13B). While alterations in the *Cdkn2a* gene are associated with NSCLC (Zhang et al., 2018), surfactant protein C is a marker of alveolar type II cells, which have been believed to be the original cell source of adenocarcinomas and NSCLC (Kwon and Berns, 2013). The down-regulated genes included paired box protein Pax-5 (Pax5; -3.6-fold; Figure 13B), a protein-coding gene whose protein expression is negative in NSCLC (Ren et al., 2015). Sclerostin domain-containing protein 1 (*Sostdc1*) also was down-regulated -4.3-fold in the *in utero* SHS plus urethane group compared to the air controls (Figure 13B). *Sostdc1* is a tumor-suppressor gene that is suppressed in NSCLC (Liu et al., 2016). This is in addition to the down-regulation of keratin 5 (*Krt5*) by -7.6-fold (Figure 13B). This gene has been identified as a down-regulated biomarker in lung adenocarcinoma, a major histological phenotype of NSCLC (Xiao et al., 2017). Overall, the histological (Figures 10, 11) and molecular data (Figure 13) provide support for our urethane-induced lung cancer model simulating features of human NSCLC.

Additionally, BALF cytology of lung cancer patients has revealed immunological responses to tumorigenesis that reflect peripheral changes in the airways (Domagała-Kulawik et al., 2003). Increased total number and percentages of lymphocytes have been found in BALF of lung cancer patients (Domagała-Kulawik et al., 2003; Hoser et al., 2003). Similarly, in our mouse model, we found mild but statistically significant increases in the percentage of lymphocytes in the BALF of the *in utero* SHS plus urethane groups at both time-points (Figures 9B,D). A report from 4 decades ago demonstrated that an antigenic stimulus could draw lymphocytes within and to the periphery of tumorigenic masses in the lungs (Ioachim et al., 1976). In our present study, tumors were observed in all groups treated with urethane (Figures 8C,F). Thus, the increase percentage of BALF lymphocytes observed in the *in utero* SHS plus urethane groups (Figures 9B,D) may be associated with an immune reaction to tumor development (Lorenz et al., 1990).

Moreover, at 48weeks of age, we found similar tumor multiplicity between *in utero* air, and SHS-urethane treated groups (Figure 8C). The average tumor volume, however, was significantly increased by the *in utero* SHS plus urethane treatment (Figure 9A). This indicates that *in utero* SHS exposure is associated with accelerated tumor growth at 48weeks of age and thus, affected late stages of carcinogenesis, namely tumor progression, rather than having an effect on tumor multiplicity, which reflects early stages of tumorigenesis (Karasaki et al., 1997). In addition, based on tumor multiplicity at both 48 and 58weeks of age, our data show that the *in utero* SHS exposure enhanced tumor development between these two time-points (Figures 8C,F; Iskandar et al., 2016). Tumor multiplicity and average tumor volume at 58weeks of age were significantly increased in the *in utero* SHS plus urethane group compared to the respective air plus urethane control group (Figures 8F, 9C). This suggests that *in utero* SHS exposure affected carcinogenesis stages associated with both tumor promotion (tumor multiplicity) and progression (tumor volume) when lung cancer development was assessed at a later time-point (58weeks of age; Karasaki et al., 1997; Wang et al., 2003; Iskandar et al., 2016). This also supports the notion that *in utero* SHS exposures impact late stages of lung cancer development. The volumetric tumor burden is a descriptive and prognostic factor that characterizes cancer progression (Mozley et al., 2010; Takenaka et al., 2016; Nishino et al., 2017; Elsayad et al., 2018). Several studies have shown that larger tumors, and thus increased tumor volumes, are associated with reduced overall survival or disease-free survival in patients (Zhang et al., 2015; Takenaka et al., 2016; Elsayad et al., 2018). Our results suggest that *in utero* SHS exposure of mice treated as adults with urethane promotes tumor growth and may be associated with a worse prognostic and decreased overall survival.

While the formation of metastases is a late step of tumorigenesis, the degradation of extracellular matrix, including basement membrane and collagen, by enzymes such as MMPs, is an early event in the progression toward tumor invasion and metastases (Hofmann et al., 2005). Here we showed significantly decreased percentages of lung collagen in the *in utero* SHS plus urethane treated mice (Figure 12), indicating changes in the protein content supporting the lung architecture. The role of MMP-12 in the lung tumor microenvironment is still poorly defined (Ella et al., 2018). Studies, however, have found elevated levels of MMP-12 in malignant lung tumors (Shah et al., 2010; Eide et al., 2016). MMP-12 expression also is elevated in NSCLC patients (Hofmann et al., 2005). This increased expression of MMP-12 also correlated significantly with relapse and local, as well as distant, metastatic processes, whereas no significant correlations were observed for MMP-1, MMP-9, MMP-10, and MMP-11 (Hofmann et al., 2005). Another recent study showed that MMP-12 mRNA levels were increased in tumor cells of lung cancer patients. This was correlated with shortened survival compared to controls (Ella et al., 2018). This correlative result between MMP-12 mRNA levels and diminished lung cancer survival was also found in other studies (Cho et al., 2004; Hofmann et al., 2005; Lv et al., 2015). Thus, substantial evidence points to MMP-12 being significantly up-regulated in human lung cancer samples.

In experimental settings, MMP-12 has been shown to promote tumorigenesis by stimulating propagation of tumor cells and tumor growth (Qu et al., 2009, 2011; Ella et al., 2018). Taken together, these reports also suggest that MMP-12, which affects the parenchymal architecture of the lungs, promotes malignancies and may be a key player involved in lung tissue extracellular matrix degradation, resulting in tumor penetration and metastasis. Since matrix metalloproteinases, *Mmp3* and *Mmp12*, as well as collagen (*Col11a1*), play key roles in extracellular matrix degradation and remodeling, dysregulated expression of these genes, as observed in our study (Figure 13), may result in lung parenchymal and structural changes, which, in turn, may favor tumorigenic progression (Shen et al., 2016; Ella et al., 2018). Our results, as evidenced by the increased number of intrapulmonary metastases (Figures 10, 11), decreased lung fibrillar collagen content (Figure 12), and up-regulation of *Mmp12* (Figure 13) in 58-week-old mice exposed *in utero* to SHS and treated with urethane, is in direct line with these studies and support previously published data. This highlights that the molecular mechanisms associated with the accelerated lung cancer progression observed in the *in utero* SHS plus urethane group at 58weeks (Figures 8–11) are associated with increased extracellular matrix remodeling (Figures 12, 13B), which promotes tumorigenesis (Shen et al., 2016; Ella et al., 2018). Thus, additionally, our study contributes to our understanding of lasting effects associated with *in utero* SHS exposures, as they identified, for the first time, *Mmp-12* gene up-regulation (9.3-fold; Figure 13) as being a key factor involved in aggravated urethane-induced lung tumor growth (Figures 8, 9) and metastases (Figures 10, 11) in 58weeks old female mouse offspring.

## Limitations

Although this study has many strengths, it also has a few weaknesses mainly associated with the selection of the animal lung disease model that was used to mimic the human pathologies of emphysema, asthma, and lung cancer. All animal disease models have advantages and drawbacks in their capacity to recreate all features of a specific human pathology. Therefore, even though no model is perfect, careful considerations of the animal model anatomy, including branching of the airways, physiology, biochemical responses and genetics, most be taken into account when selecting a model (Williams and Roman, 2016). For the emphysema study, we selected the elastase-induced mouse model, since it is based on the proteases-anti-proteases imbalance paradigm of emphysema pathogenesis and is widely used and accepted in the scientific community to reproduce key features of the human pathology, including inflammation, oxidative stress, declining lung function and enlarged airspace in a cost-and time-efficient manner (Antunes and Rocco, 2011). For the asthma study, although allergic airway diseases are complex and have many types of clinical manifestations, we selected the HDM-induced mouse model as it is well-known that BALB/c mice in response to sensitization and challenge to an allergen such as HDM, exhibit marked Th2 immune responses, lung eosinophilic inflammation, as well as airway hyperresponsiveness (Shin et al., 2009). Thus, this model recapitulates a significant set of hallmark characteristics of asthmatic responses. For the lung cancer study, we used the highly reproducible urethane-induced lung cancer mouse model to simulate the time-dependent progression of lung tumor development from hyperplasia, adenoma, adenocarcinoma, to metastasis, the same sequence of events seen in human lung cancer (Kellar et al., 2015). We used three distinct ‘chemically-induced’ models in healthy BALB/c mice to recreate emphysema, asthma, and lung cancer pathogeneses. This is in contrast to genetically modified animal models, including knockout mouse models, which are useful preclinical models, since they allow for the study of the effect of a specific gene on the development of a particular disease. Our laboratory is currently investigating the interaction between *in utero* SHS exposures – as well as exposures to tobacco-related products (e.g., electronic-cigarettes) – and the MMP12 gene in MMP12 knockout mice. This mouse model, with the MMP12 gene absent, should enable isolation and more precise determination of the role that MMP12 plays in this particular *in vivo* experimental context. Thus, extrapolation of the findings described here from mice to humans needs to be made cautiously and with the caveat that the mechanisms of disease and the implications of the biological responses observed are based on chemically induced animal models of diseases.

## Conclusion

A better understanding of the contribution of *in utero* SHS exposures to the epigenetic events and molecular mechanisms affecting fetal lung programming and subsequent post-natal lung development should help target potential effective measures to prevent the development of significant adult lung diseases. This study showed that *Mmp12* in adults is upregulated by *in utero* SHS exposures and is a crucial factor contributing to aggravated lung responses in adult emphysema, asthma, and lung cancer mouse models. Thus, our results in mice support the relationship between *in utero* SHS exposures and *Mmp12* up-regulation. Further, these results bring attention to and increase the awareness of, *in utero* SHS as an underestimated but yet critical risk factor for the development of emphysema, asthma, and lung cancer in adulthood.

## Data Availability Statement

The original contributions presented in the study are included in the article. The RNA sequencing data presented in the study are deposited in the Mendeley Data repository, available at https://data.mendeley.com/datasets/rbwcr4srkb/1. DOI 10.17632/rbwcr4srkb.1. Further inquiries can be directed to the corresponding author.

## Ethics Statement

The animal study was reviewed and approved by Louisiana State University Institutional Animal Care and Use Committee.

## Author Contributions

AN and AP designed the studies. AN, ZP, RX, HH, VD, KL, SS, and DP carried out the assays. Also, MG and AP assisted with data analysis. The manuscript was written by AN and revised by AP. All authors approved the final manuscript.

## Funding

This study was supported by a grant (AP) from the Louisiana Governor’s Biotechnology Initiative GBI-BOR#013. At the time the studies were initiated, AN was a recipient of a post-doctoral fellowship from the Fonds de Recherche Québec Santé (FRQS). MG is supported by National Science Foundation (NSF CAREER award number: 2045640).

## Conflict of Interest

The authors declare that the research was conducted in the absence of any commercial or financial relationships that could be construed as a potential conflict of interest.

## Publisher’s Note

All claims expressed in this article are solely those of the authors and do not necessarily represent those of their affiliated organizations, or those of the publisher, the editors and the reviewers. Any product that may be evaluated in this article, or claim that may be made by its manufacturer, is not guaranteed or endorsed by the publisher.
